# Network-guided prediction of aromatase inhibitor response in breast cancer

**DOI:** 10.1371/journal.pcbi.1006730

**Published:** 2019-02-11

**Authors:** Matthew Ruffalo, Roby Thomas, Jian Chen, Adrian V. Lee, Steffi Oesterreich, Ziv Bar-Joseph

**Affiliations:** 1 Computational Biology Department, School of Computer Science, Carnegie Mellon University, Pittsburgh, Pennsylvania, United States of America; 2 Women’s Cancer Research Center, Department of Pharmacology and Chemical Biology, UPMC Hillman Cancer Center, Magee Womens Research Institute, Pittsburgh, Pennsylvania, United States of America; 3 Machine Learning Department, School of Computer Science, Carnegie Mellon University, Pittsburgh, Pennsylvania, United States of America; Memorial Sloan-Kettering Cancer Center, UNITED STATES

## Abstract

Prediction of response to specific cancer treatments is complicated by significant heterogeneity between tumors in terms of mutational profiles, gene expression, and clinical measures. Here we focus on the response of Estrogen Receptor (ER)+ post-menopausal breast cancer tumors to aromatase inhibitors (AI). We use a network smoothing algorithm to learn novel features that integrate several types of high throughput data and new cell line experiments. These features greatly improve the ability to predict response to AI when compared to prior methods. For a subset of the patients, for which we obtained more detailed clinical information, we can further predict response to a specific AI drug.

## Author summary

Breast cancer is the second most common type of cancer in women, with an incidence rate of over 250,000 cases per year, and breast cancer cases show significant heterogeneity in clinical and omic measures. Estrogen receptor positive (ER+) tumors typically grow in response to estrogen, and in post menopausal women, estrogen is only produced in peripheral tissues via the aromatase enzyme. Inhibition of aromatase is often an effective treatment for ER+ tumors, but aromatase inhibitor therapy is not effective for all tumors, and causes of this heterogeneity in response are largely not known. In this work, we present a feature construction and classification method to predict response to aromatase inhibitor therapy. We use network smoothing techniques to combine tumor omic data into predictive features, which we use as input to standard machine learning algorithms. We train predictive models using clinical data, including high-quality clinical data from UPMC patients, and show that our method outperforms previous approaches in predicting response to aromatase inhibitor therapy.

## Introduction

A number of recent large efforts have focused on collecting genomic data from tumors. While these datasets led to several successful studies and insights, in many cases the clinical data available for patients enrolled in these studies is incomplete. This makes it hard to use such datasets for predicting tumor specific outcomes and tailoring treatments to individuals.

To develop accurate methods for for predicting treatment responses we need both, a comprehensive genomic dataset profiling the individuals being studied and accurate complimentary clinical information. To date, methods that used the former (detailed genomic data) usually were unable to use the latter for a significant number of individuals while methods that only relied on clinical information are limited in their ability to distinguish between tumor responses [1].

Consider, for example, the genomic data that is part of The Cancer Genome Atlas (TCGA, [2]). Several methods have used this data to study general questions related to cancer biology and prognosis. Examples include methods to identify molecular targets for cancer therapy [3], enhancement / creation of general prognostic classification systems [4–6], *de novo* pathway identification via identification of mutually exclusive mutations [7] and identification of genes implicated in cancer via combinations of different data types [8]. In contrast, most efforts for predicting response to specific treatments have been limited to much smaller datasets, usually focused only on specific pathways or classes of mutations, and often only relying on *in vitro* (cell line) experiments which have limited clinical utility [9–11]. Indeed, in many cases a key challenge researchers face when trying to predict such specific response is the lack of detailed and well-curated clinical data to supplement the high throughput molecular data in the large databases.

Here we focus on response to aromatase inhibitors (AIs), which block the conversion of androgen to estrogen and thus lower systemic estrogen. AIs show superior efficacy for the treatment of postmenopausal ER+ breast cancer compared to tamoxifen [12]. Despite the significant reduction of recurrence, resistance is common, and remains a tremendous clinical and societal problem. Mechanisms of resistance are very heterogenous [13], and it is currently not possible to accurately predict response for specific AI treatments. Thus, methods for predicting tumor specific AI responses are urgently needed, especially given availability of choices of endocrine therapy, their potential side effects, and recent findings that extended endocrine treatment benefits a subset of patients [14].

To predict AI response we developed computational methods to construct network smoothed features based on breast cancer genomic data from the Cancer Genome Atlas (TCGA) and combined these with manually curated clinical data for a subset of patients in TCGA that were treated at the University of Pittsburgh Medical Center (UPMC). Many previous approaches have been developed to integrate multiple types of omic data using a variety of techniques: multiple kernel learning [15–18], joint matrix factorization [19, 20], latent variable models [21, 22], and other network-based data integration methods [23, 24], though most of these methods have drawbacks in treatment-specific prediction tasks. Such methods are typically either unsupervised, and therefore intended for general-purpose clustering and stratification of patients, or sacrifice genomic / clinical interpretability.

The UPMC clinical data included information on the treatment patients received, its effectiveness and the outcomes. The genomic data we used included sequence variations, expression changes and cell line drug responses all smoothed using general protein-protein interaction networks. We used the clinical and genomic features to predict treatment response and overall survival. Overall we show that by combining genomic and clinical attributes we can obtain high accuracy and predicting cancer survival and slightly improve this accuracy when incorporating functional cell line data. For the more challenging task of predicting treatment outcome we show that both, the addition of the cell line data and the improved clinical data, leads to greater accuracy and improves upon prior methods.

## Results

To predict response to aromatase inhibitors we combine high throughput gene expression and sequencing data with detailed clinical data for individual patients (Materials and methods). While using the expression, mutation and interaction data directly in a prediction framework can sometimes lead to useful results, as we show below in many cases such data is too sparse to provide useful features given the (relatively small) number of patients. Thus, a major challenge in the construction of successful prediction methods is learning useful summary features from the high throughput data. Here we use network smoothing to combine expression, mutation, protein interaction and drug response data across tumors and cell lines. These networks are then converted to PCA components which can be computed for each tumor and summarize the tumor expression and mutation information using the protein interaction network. These components, together with the clinical data are then used as features for several different classifiers we tested. Using these features and the labels obtained from the clinical records, we perform cross-validation experiments to examine our ability to predict non-response to aromatase inhibitor treatment.

**Fig 1:**
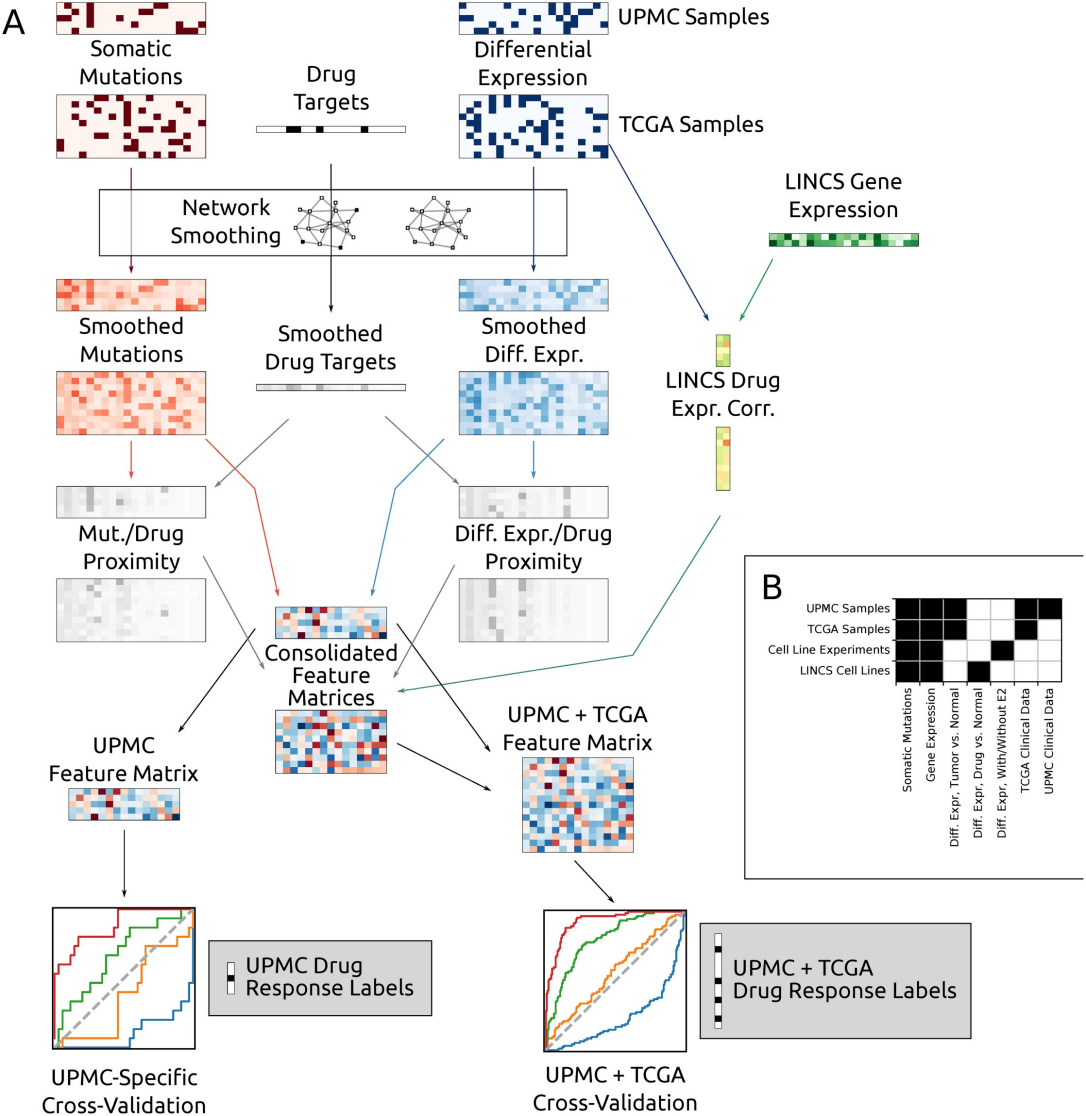
**(a)** Flowchart of our general classification approach, showing the network smoothing procedure applied to multiple data types: somatic mutations, differentially expressed genes, and protein targets for a particular drug. Smoothed mutations, differential expression, and drug targets are combined into network proximity measures by computing the element-wise minimum of the smoothed scores. Correlation is computed between LINCS expression profiles and tumor gene expression measurements. UPMC and TCGA samples are handled identically for most of the analysis pipeline until performing cross-validation: UPMC samples are used both in isolation and in combination with TCGA samples. **(b)** shows feature availability for data types used in this analysis.

Our feature construction workflow is shown in Fig 1a, which demonstrates the combination of genomic data into predictive features for UPMC samples, TCGA samples, and LINCS cell lines. Fig 1b shows the availability of features for each data source used in our analysis.

While the specific genes mutated in a pathway may vary between tumors, they are likely close in the graph representing the interaction network. A graph smoothing method tries to find mutated pathways by allowing information to propagate along edges in the graph and then finding sub-graphs (a collection of connected nodes) that are weighted highly. These subgraphs represent a set of genes that are mutated in several tumors and so are useful for predicting clinical outcomes for this set of patients. Smoothing is a general strategy and here we use it to combine several different types of genomic data including mutation and expression changes in tumors and drug response profiling in cancer cell lines. To summarize the smoothed networks in a few components (features) we perform PCA decomposition on the matrix obtained across the tumors for each data type (mutations, expression and drug targets). See Materials and methods for complete details.

### Combined prediction model outperforms individual constructed features

We first tested our method on the set of 590 breast cancer TCGA samples that were either prescribed aromatase inhibitors, or were not considered for this type of treatment given their ER status, for which we assigned artificial “non-response” labels (see Materials and methods, Classification). Figure A in S1 Text shows the univariate predictive performance of individual features we used based on ROC AUC metric, showing the top 20 and bottom 20 features sorted by AUC. As can be seen, while none of the features provide very high accuracy on their own (the best single feature is the mean across all genes of min{protein targets of arimidex, smoothed differential expression} with an AUC of 0.81), several features are still informative in isolation. Overall, the best single features are those using the PCA decomposition of the expression data and those that combine expression and drug target information (protein targets of aromasin and gene targets of estrogen receptors). We also see that the drug targets features from LINCS (Methods) related to Arimidex are only weakly informative which may indicate that the specific cell line used for this drug (HA1E, kidney) is not enough for extracting general drug response profile for Arimidex.

**Fig 2:**
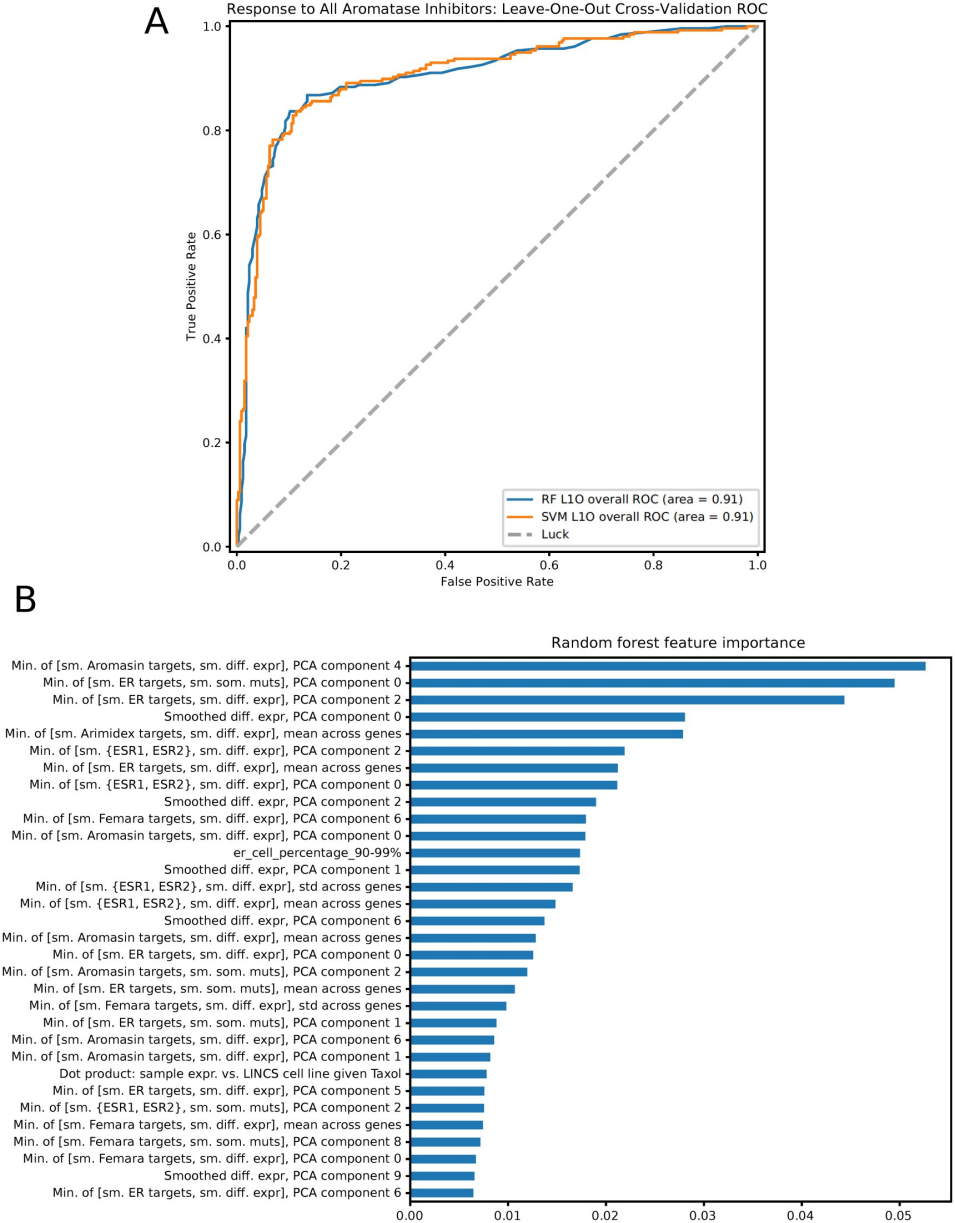
**(a)** Leave-one-out cross-validation results for prediction of non-response to all aromatase inhibitors, using random forests and probabilistic support vector machines. **(b)** Feature importance from the random forest cross-validation results, showing which constructed features contribute most to the random forest fit. Features prefixed with “Min.” denote elementwise minimum of pairs of matrices, *e.g.* smoothed (“sm.”) drug targets of Arimidex and smoothed binary differential expression as shown in the first feature listed. “sm. {ESR1, ESR2}” denotes network proximity to the *ESR1* and *ESR2* genes. Sample×gene matrices are collapsed across genes in various ways to produce feature values for samples: mean or standard deviation across all genes, or through PCA decomposition. Categorical clinical features are represented with one-hot encoding, and are shown as “feature name_column name”, *e.g.* “er_cell_percentage_90-99%”.

We next trained classifiers using all features to predict general response to aromatase inhibitors. Fig 2a shows cross-validation performance in prediction of aromatase inhibitor response, using probabilistic SVM and Random Forest (RF) classifiers, the top two performing methods among those we tested (See Supplement for the performance of the other classifiers). We see that both classification methods lead to high mean ROC AUC (0.91), demonstrating the advantage of integrating several different types of features. While both methods performed equally well, it is much easier to interpret the RF results and so we focus on these results below. Fig 2b shows the importance of each feature used by RF (using the scikit-learn package [25]). Again, features that combine tumor expression changes with drug target information seem to be the most useful including features based on estrogen receptor targets and targets of aromasin. We also find a high scoring feature that combines tumor mutation information with estrogen receptor target information. Specific genes contributing to these high scoring PCA features are plotted in Figures B, C, and D in S1 Text. These genes include *TP53* whose mutations were identified as most significant contributors for the top feature, in line with a recent study by Gellert *et al.* [26], in which *TP53* mutations were associated with poor response in tumors treated with AIs. Other top genes included *CDH1*, which is involved in cancer progression and metastasis [27], *JUN*, a transcription factor implicated in cell proliferation and angiogenesis in invasive breast cancer [28], and *KLK4*, which codes for a kallikrein protein that is overexpressed in prostate cancer [29] and is associated with an epithelial-mesenchymal transition-like effect in prostate cancer cells [30].

### Cell line results improve prediction performance

**Fig 3:**
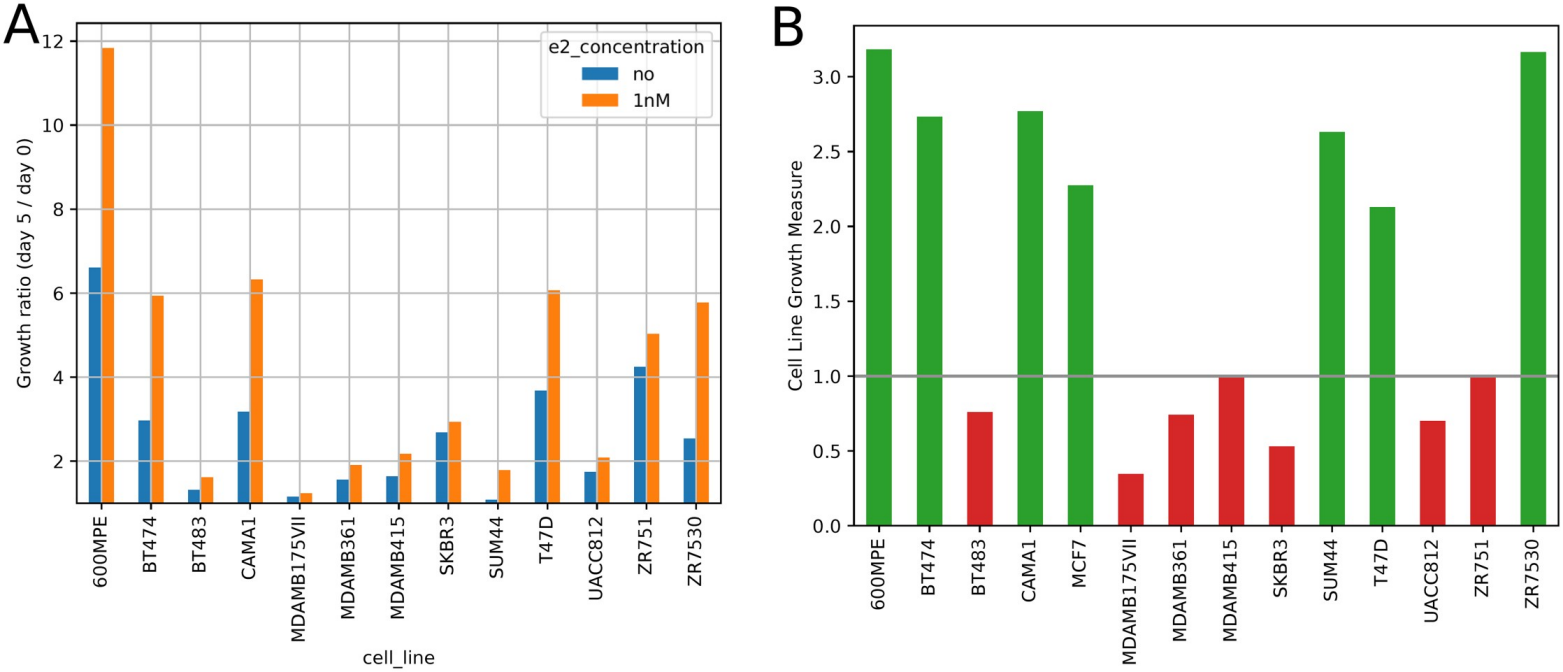
Cell line growth data. **(a)** shows cell line growth ratios (day 5 cell count / day 0 cell count), with and without 1nM serum estrogen. **(b)** shows the cell line growth measure defined in Eq 6. Threshold 1.0 was used to denote cell lines as responsive (green) or non-responsive (red).

To obtain additional data for improving the ability of our method to predict tumor response to aromtase inhibitors we performed cell line experiments. In these experiments we grew a selection of ER+ and control ER- breast cancer cell lines in serum estrogen for 5 days and then either kept the estrogen-containing serum for an additional 5 days, or switched to serum free media, thus mimicking the removal of estrogen. Growth measure results for these cells are presented in Fig 3. As can be seen, for several cells there are significant differences with and without serum estrogen. Since, unlike for the patients, we only have genomic data and no clinical information for these cells, we developed a joint prediction method by combining tumor and cell line derived classifiers (Materials and methods, Combining cell line and patient derived classifiers). The joint prediction combined the predictions of the two separate classifiers (tumor and cell line based) by learning a weight for each of them. As expected, given the small number of the cell lines tested compared to the number of patients (13 vs. 590), the weight assigned to the cell line predictor was lower (median *γ* = 0.0276, mean *γ* = 0.0286 across all leave-one-out cross-validation folds for random forest classifiers). As can be seen in Figure Q in S1 Text, with curves in the legend sorted by AUC, the addition of cell line information slightly improves cross-validation performance (though this difference is not visible when limiting AUC values to two decimal places).

### UPMC clinical data improves prediction of anastrozole response

**Fig 4:**
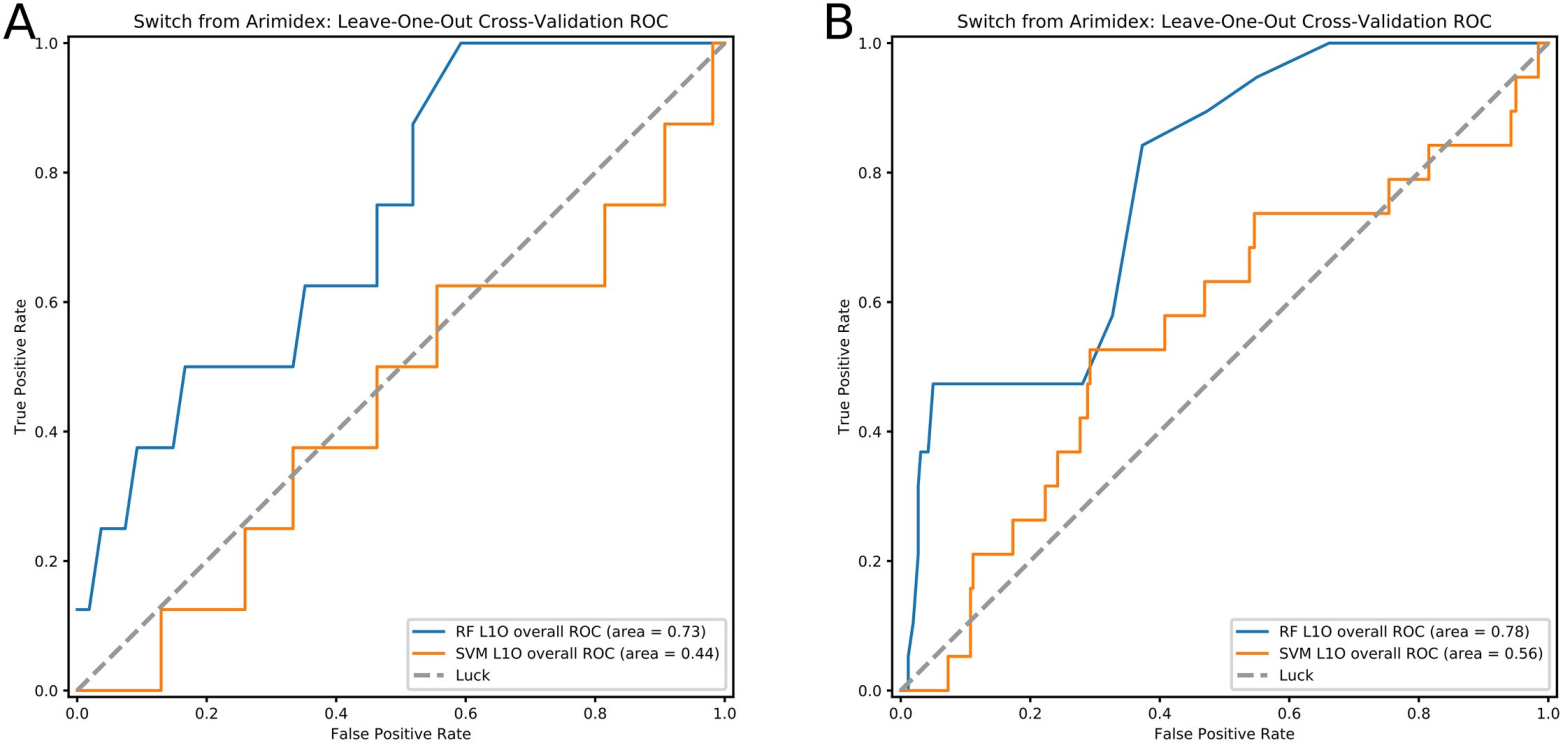
Cross-validation prediction results for non-response to anastrozole. **(a)** shows results for non-response to anastrozole with all available TCGA samples, and **(b)** shows prediction results restricted to UPMC patients.

The cross-validation results presented above correspond to an overall prediction of whether a tumor responds to an aromatase inhibitor. In the “real world”, patients receive one out of three AIs, and within our cohort Arimidex (anastrozole) was the most frequently prescribed drug. Given some differences in mechanism of action, side effects and efficacies [31–33], we next used our method to predict response to Arimidex. Results are shown in Fig 4a. While this is a much more challenging prediction task than overall response to AIs (reflected in the decreased overall accuracy) the results still show the predictive power of the features that we compute. These results can be further improved with better clinical data.

The discussion so far focused on all TCGA breast cancer samples. For a subset (*n* = 151) of these patients we also have high-quality, manually-curated patient data, allowing us greater accuracy in identifying and predicting clinical outcomes (a detailed list of the clinical variables we extracted for this cohort is available on the supporting website). We thus examined the difference in performance between training and testing on all TCGA patients and using only University of Pittsburgh/UPMC patients (*n* = 62 given anastrozole, and *n* = 89 which were ER– or given any aromatase inhibitor). Results from this analysis are shown in Fig 4b and Figure I in S1 Text. While we do not observe a large performance difference when predicting response to all aromatase inhibitors (indicating that TCGA clinical features for such analysis are likely good enough), we see a larger improvement when predicting of response to anastrozole alone (ROC AUC 0.73 for UPMC samples compared to 0.70 for all TCGA samples). This indicates that accurate information about the specific drugs used for each patient, switching between drugs and responses and side effects, all present in the UPMC curated data but not in the TCGA data, can greatly help automated methods for feature construction in personalized medicine analysis.

### Comparison with other methods

**Fig 5:**
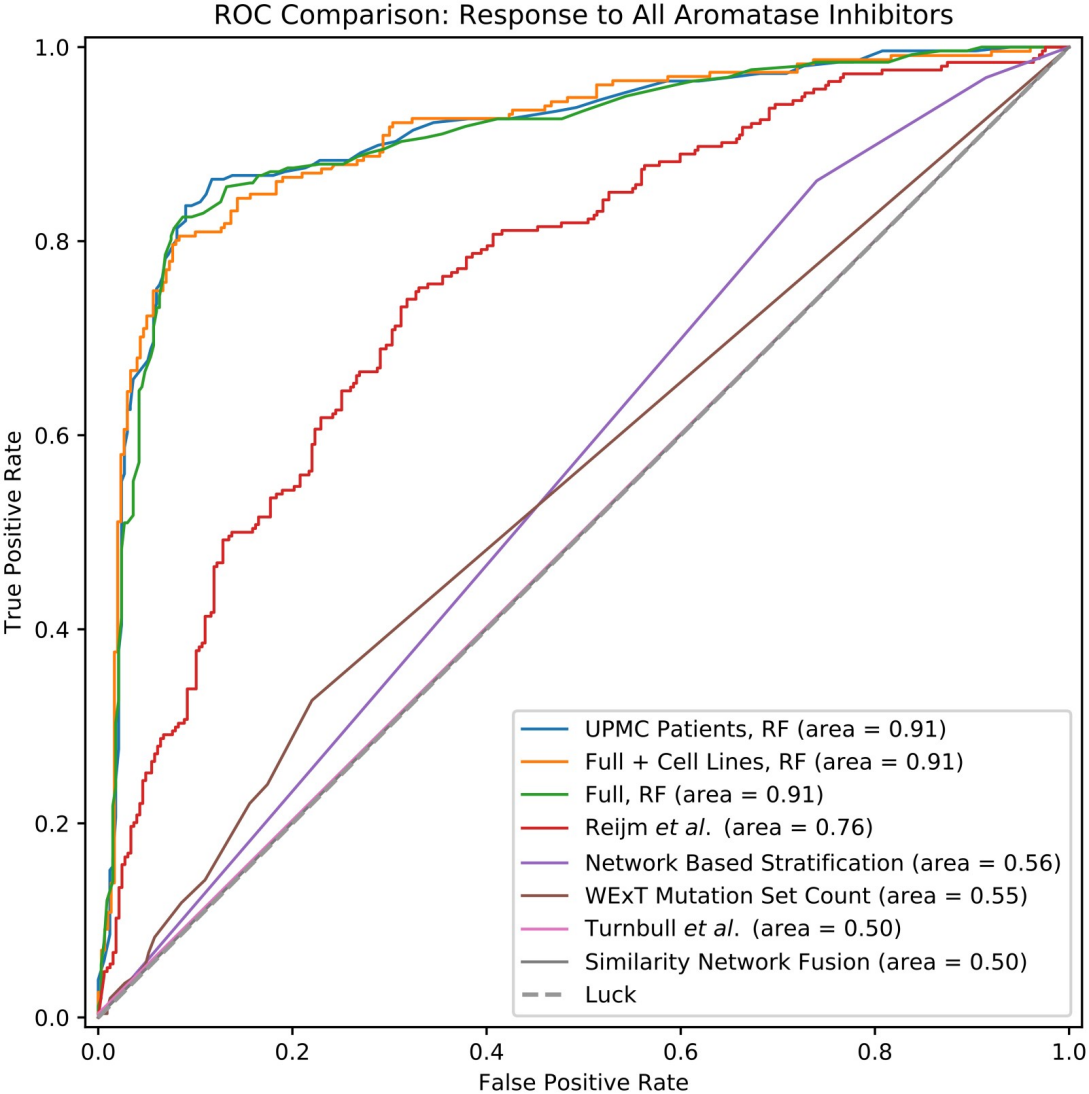
Performance comparison between multiple prediction strategies, for prediction of aromatase inhibitor non-response.

To evaluate the usefulness of the features we constructed for this prediction task we first compared the results of using these features to methods that only use the measured expression and sequence data [34–39]. For this we constructed a “naïve” feature set, consisting only of somatic mutations, differential expression, and binary indicator columns for the clinical features (Methods). We repeat our cross-validation analysis using this feature set, using the “raw” binary features, and using the top 8, 32, 128, and 512 components from PCA decomposition/transformation of this matrix. Results are shown in Fig 5, Figure E in S1 Text and Figure G in S1 Text for all aromatase inhibitors, and Figures F, H, and R in S1 Text for anastrozole. We see that performance of these ‘naïve” feature is comparable for the “all aromatase inhibitor” case (leave-one-out ROC AUC 0.90 for binary features, max 0.90 for PCA decomposition, vs. 0.91 for our constructed feature set), while it is significantly lower for the more challenging task of predicting response to anastrozole (leave-one-out ROC AUC 0.59 for binary features, 0.62 for PCA decomposition, vs. 0.70 for our constructed feature set). We also repeated the University of Pittsbugh/UPMC-only analysis with this ‘naïve” feature set, and again note a large drop in performance (with ROC AUC dropping from 0.70 to 0.44 for anastrozole). See Figures J and K in S1 Text for full results.

We also compared our method to prior methods that used either a network based approach to analyze mutation data [40], relied on mutually exclusive mutations [7] for prognosis classification, or combined disparate network similarity measures across networks [23]. Results are presented in Fig 5 and Figure R in S1 Text. In general we find that such methods, which only use mutation information, do not perform as well as our methods that integrate several different types of data including expression and drug targets.

We have also compared our method to prior methods that are specifically focused on predicting AI response. Turnbull *et al.* [41] performed feature selection on gene expression data resulting in four genes which were used in a decision tree framework to predict tumor response to AIs. We reimplement their decision tree classifier (Supporting Methods) and used it for response predictions. Results are shown in Fig 5 for all aromatase inhibitor prediction, and Figure R in S1 Text for specific prediction of anastrozole non-response. The low accuracy of this method is likely due to the specific parameters used in the original study that are likely not appropriate for a the larger dataset studied in this paper. See Supporting Results for more details. Reijm *et al.* [42] developed an eight-gene classification system for prediction of AI response, and presented *t*-statistic values for association of these genes with tumor response. Though there are conceptual differences between *t*-statistic values and logistic regression coefficients, we nonetheless can use these *t*-values to produce continuous predictions of tumor response with log-fold gene expression data (Supplementary Methods). Results for this method are presented in Fig 5 and Figure R in S1 Text. We see reasonable performance for prediction of aromatase inhibitor response (ROC AUC 0.76) though substantially worse performance for anastrozole-specific response (ROC AUC 0.53).

## Discussion

We combined clinical and high throughput patient data with additional cell line experiments to predict tumor response to aromatase inhibitors. We developed methods for constructing PCA features by smoothing interaction networks overlaid with expression, mutation and drug target data. Our clinical data consisted of abundant (though less accurate) data for all TCGA patients and from a more detailed curated dataset for a subset of 151 patients in this set.

To further improve the classifiers and the labels, we analyzed electronic medical records within the University of Pittsburgh Medical Center (UPMC) system for the subset of TCGA patients that were treated at UPMC. These elements constituted data involving known breast cancer risk factors that either were not included in the TCGA data sets, or it was uncertain how the data was obtained and/or validated. This included reproductive history of patients at the time of breast cancer diagnosis, family history including both first and second degree relatives as well as other malignancy history for the patients if applicable. Data involving comorbid diseases that are common in adult populations including hypertension, diabetes, hyperlipidemia and metabolic information on patient’s weight at the time of diagnoses were also obtained, as these may impact patients’ breast cancer specific survival as well as overall survival. In regards to tumor biology, information from the EMR was obtained to the specific degree of hormone receptor status including H-score or percent staining as well as HER2/neu status. As we show, the models we developed provide accurate general predictions for the success of treatment with aromatase inhibitors. Focusing on treatment with a specific drug, Arimidex, we show that using the more detailed clinical data can lead to much better results when using our methods, greatly improving upon the use of naïve features and on prior methods suggested for this task.

For our labels, the analysis of EMRs allowed us to obtain the most up to date survival data. While most TCGA based analysis relies on survival data that was collected several years ago, EMRs are continuously updated and so we were able to use much more up to date information. Finally, we were able to use the EMRs to determine reasons for stopping specific therapy or drug including toxicity. Combined, the new features and improved labels, led to better performance for the challenging task of predicting response to a specific drug as we showed in Results.

The top-scoring PCA component in the random forest prediction is strongly influenced by the cell cycle gene *CCND1*, overexpression of which correlates with early cancer onset and tumor progression [43, 44]. It has been well known that proliferation is a strong predictive factor of endocrine treatment response, for example elegantly shown in a series of neoadjuvant short-term pre-surgical studies [45–47]. Another gene that ranked highly in our feature importance score was *CDH1*. *CDH1* encodes E-cadherin, a calcium-dependent cell-cell adhesion protein, that is frequently mutated in a number of tumor types, including breast cancer. E-cadherin protein is lost in up to 95% of invasive lobular breast cancer, and is one of the hallmark features of this disease, whereas it is lost in less than 5% of invasive ductal breast cancers [48]. We have previously shown that estrogen treatment of breast cancer cells results in downregulation of E-cadherin, potentially contributing to estrogen-mediated activation of migration and motility of cells [49]. In addition, we have shown that ILC cell lines with genetic loss of *CDH1* have a unique estrogen response compared to IDC cell lines [50]. Thus the results from this study further suggest a critical role for E-cadherin in response to estrogen and aromatase inhibitors.

Much prior work has focused on the prediction of response to endocrine therapy in general and aromatase inhibitors specifically [10, 51–53]. However, only a few of these studies are directly comparable to this work. Many prior studies use data types unavailable for large clinical datasets (*e.g.* proteomic data), focus on other organisms such as mice, or are restricted to cell lines only. We therefore focused on comparison to relevant work and on the analysis of the usefulness of the features constructed. As we show, for the more challenging task of predicting specific drug response our method outperforms especially when comparing the results for the more accurate the University of Pittsburgh/UPMC cohort.

Our results indicate that, while high throughput datasets are key to constructing accurate prediction methods, it is extremely important to couple these datasets with complete and accurate clinical data. While information on drug prescription and usage is available for all individuals in the TCGA breast cancer dataset, we found several discrepancies between the more detailed UPMC data and the TCGA data for the same individual. This may indicate that data on other patients is noisy as well. We believe that our study provides a strong incentive for additional efforts aimed at curation of such clinical data.

## Materials and methods

### Data

The input to our method consists of genomic and clinical BRCA (breast cancer invasive carcinoma) data obtained from TCGA [54] and detailed clinical data for a subset of 151 University of Pittsburgh/UPMC patients (data description on supplementary website). The UPMC data provides specific treatments, reasons for changes in treatments, dates and responses for these patients. Specifically, we used the following data types:

- Somatic mutations obtained from whole-exome sequencing.
- Gene expression data, postprocessed and provided as part of the COSMIC cancer gene census [55], as both continuous log-fold change and binary differential expression status.
- LINCS [56] gene expression signatures for the cell lines treated with the drug we studied.
- Treatment data available in TCGA clinical information: drugs that each patient was prescribed, and global “responded to treatment” status for the patient’s entire drug regimen.
- Treatment data parsed by University of Pittsburgh/UPMC researchers which in addition to the TCGA information mentioned above includes detailed clinical information about dates of specific events and reasons for patients who discontinued the use of a specific drug.

We focus on the three aromatase inhibitors prescribed most in BRCA patients: anastrozole (Arimidex), exemestane (Aromasin), and letrozole (Femara). To construct labels for tumors (response / non response) for each of these drugs, we examine the treatment information to identify which patients were prescribed that drug, and whether the patient discontinued that drug due to non-response. We then construct a “non-response” vector for each drug, denoting a patient as positive if the patient discontinued that drug or died during treatment with it.

### Pre-processing the omics data

We constructed a binary mutation matrix *M*, a log-fold gene expression matrix *E*, and a binary differential gene expression matrix *D*, with samples as rows and genes as columns. We use *C*(*A*) to denote the set of column labels of matrix *A*, so that *e.g.* *C*(*M*) is the set of genes that appear in the TCGA somatic mutation data. Similarly, we define *R*(*A*) as the set of row labels of matrix *A*, corresponding to the distinct samples (individuals) present in each data set.

The mutation matrices *M* are defined as

$$\begin{array}{c} M\left[i,j\right]=\{\begin{array}{cc} 1 & \text{if}\;\text{gene}\;j\;\text{is}\;\text{mutated}\;\text{in}\;\text{sample}\;i,\\ 0 & \text{otherwise} \end{array} \end{array}$$ (1)

The COSMIC database [55] provides differential gene expression data for TCGA samples, represented as log-fold change between tumor and matched normal samples in the same tumor/tissue. The COSMIC database additionally annotates each log-fold differential expression measurement with “over”, “under”, or “normal” gene expression, for genes with log-fold differential expression outside *σ* = 2 standard deviations from the mean in each sample. We collect the continuous log-fold gene expression measurements into a matrix *E*, and collect the normal/over/under expression status into a binary matrix *D*:

$$\begin{array}{c} D\left[i,j\right]=\{\begin{array}{cc} 1 & \text{if}\;\text{gene}\;j\;\text{is}\;\text{over-}\;\text{or}\;\text{under-expressed}\;\text{in}\;\text{sample}\;i,\\ 0 & \text{otherwise} \end{array} \end{array}$$ (2)

The TCGA BRCA data includes somatic mutations in 22,232 genes across 1,081 samples, and differential expression for 17,747 genes in 1,079 samples.

In addition to the condition specific omics data we also use general interaction datasets. We use the HIPPIE protein-protein interaction network [57, 58] (version 2.1, released 2017-07-18), which contains confidence scores for 318,757 interactions between 17,204 proteins. Additionally, we use gene expression data from the LINCS LDS-1191 assay, which contains measurements of gene expression in cell lines after gene knockouts and introduction of small molecules (“perturbagens”). We use gene expression data in cell lines given the chemotherapy agent Taxol, and the aromatase inhibitor Arimidex.

### Gene set network smoothing

As has been shown in the past, protein interaction networks provide a useful way to overcome data sparsity and noise when predicting cancer responses [4]. Here we use the network propagation/smoothing method described in Vanunu *et al.* [59] to combine omics data across patients. Given a network *G* = (*V*, *E*, *w*) with *V* as the set of proteins, *E* as the set of their interactions, *w*(*u*, *v*) representing the reliability of an interaction (*u*, *v*)∈*E*, and a prior knowledge vector *Y*: *V* → [0, 1], we compute a function *F*(*v*) ∀*v* ∈ *V* that is both smooth over the network and accounts for the prior knowledge about each node.

This network smoothing process uses a normalized edge weight matrix *W*′, computed via Laplacian normalization of the edge weight matrix *W*: we first construct a diagonal matrix Δ with Δ[*i*, *i*] = ∑_*j*_ *W*[*i*, *j*], and compute *W*′ = Δ^−1/2^ *W*Δ^−1/2^. Given a prior knowledge vector *Y*, we then compute the smoothed vector *F* using the iterative procedure described by Zhou *et al.* [60]. Starting with *F*^(0)^ = *Y*, we update *F* at iteration *t* as follows:

$$\begin{array}{c} F^{\left(t\right)}=\alpha W^{\prime }F^{\left(t-1\right)}+\left(1-\alpha \right)Y \end{array}$$ (3)

This procedure is repeated iteratively until convergence; namely we stop when ‖*F*^(*t*)^−*F*^(*t*−1)^‖_2_ < *ϵ*. Note that Laplacian normalization produces a *W*′ with |λ|_max_ ≤ 1, which is required for this iterative method to converge.

When *Y* is a binary vector, *i.e.* *Y*[*u*]∈{0,1}∀*u* ∈ *V*, the value *F*[*v*] for a gene *v* in the smoothed vector *F* naturally corresponds to a continuous measure of network proximity between *v* and the “selected” genes *s* ∈ *S* ⊆ *V* for which *Y*[*s*] = 1. We therefore this network smoothing method to compute scores of proximity for each gene with respect to multiple gene sets:

- For each tumor, genes in which that tumor harbors a non-synonymous somatic mutation.
- Differentially expressed genes in each tumor, from the COSMIC cancer gene census [55].
- Protein targets of breast cancer drugs, from queries to DGIdb [61].
- Estrogen receptor proteins ESR1 and ESR2.
- Genes targeted by (transcription factors) ESR1 and ESR2, as listed in the TRRUST database [62].

For each aforementioned gene set *S*, we construct a binary prior knowledge vector *Y*_*S*_:

$$\begin{array}{c} Y_{S}\left[s\right]=\{\begin{array}{cc} 1 & \text{if}\;s\in S\cap V,\\ 0 & \text{otherwise} \end{array} \end{array}$$ (4)

We then perform network propagation on the vector *Y*_*S*_, producing a vector *F*_*S*_. Note that not all genes in the set *S* are necessarily included in the protein interaction network, and therefore the vectors *Y* for *e.g.* somatic mutations in a tumor can differ from rows of the somatic mutation matrix *M*.

For somatic mutations and differential expression, we then collect the smoothed vectors into “propagated” matrices *M*_*P*_ and *D*_*P*_, with *R*(*M*_*P*_) = *R*(*M*) = *R*(*D*_*P*_) = *R*(*D*) and *C*(*M*_*P*_) = *C*(*D*_*P*_) = *V*. Intuitively, the propagated matrices *M*_*P*_ and *D*_*P*_ contain the per-sample binary vectors of *M* and *D* smoothed over the network. In biological terms, each row of these matrices represents the network proximity of each gene product to mutated and differentially expressed genes in that sample. Consequently, as illustrated in Fig 1, the columns of these matrices provide propagated mutation and differential expression profiles for each gene product across all samples, indicating the proximity of the respective gene product to the products of mutated or differentially expressed genes in the respective sample.

### Network-integrated proximity features

We next combine the smoothed matrices *M*_*P*_ and *D*_*P*_ with the smoothed vectors of multiple gene sets *S* as mentioned above:

- Proteins targeted by anastrozole
- Proteins targeted by exemestane
- Proteins targeted by letrozole
- Estrogen receptor proteins
- Genes targeted by estrogen receptors

Protein targets of each drug are obtained from queries to DGIdb [61], and gene targets of estrogen receptors are obtained from the TRRUST database [62].

Given one of the smoothed “target” vectors described above, denoted as *T*, we compute a new matrix *M*_*P*,*T*_:

$$\begin{array}{c} M_{P,T}\left[i,j\right]=\min\{M_{P}\left[i,j\right],T\left[j\right]\} \end{array}$$ (5)

That is, for some tumor *i* and gene *j*, the value *M*_*P*,*T*_[*i*,*j*] quantifies gene *j*’s proximity to both somatic mutations in tumor *i* and the gene set *S* represented by the smoothed vector *T*. We compute *D*_*P*,*T*_ similarly, replacing *M*_*P*_ with *D*_*P*_ in Eq 5.

We use these matrices to compute features for response to treatment in tumor *i*:

- Row-wise mean, representing the total network proximity between a tumor’s somatic mutations (or differential expression) and genes in a predefined set (*e.g.* targets of a specific drug).
- Row-wise standard deviation, quantifying the variance of mutational or differential expression proximity to drug targets.

In addition to these summary statistics for each tumor, we also perform PCA decomposition of these “minimum” matrices *M*_*P*,*T*_ and *D*_*P*,*T*_, and use the top 10 PCA components as predictive features. Plots of PCA component scores for genes are shown in Figures B, C, and D in S1 Text—in these figures, genes are sorted by absolute value of there scores as assigned by PCA decomposition, and these absolute values are plotted as the importance of each gene for that PCA component.

### LINCS expression features

We obtained data from the LINCS project [56] L1000 LDS-1191 assay, which has profiled the gene expression of many cell lines under normal conditions, after introduction of small molecules (“perturbagens”), and under gene knockouts. We selected the experiments involving the drugs analyzed in this study and identified the DE genes for each of these treatments.

Two relevant drugs have been administered to cell lines by the LINCS consortium: Taxol (a taxane, also known as Paclitaxel and Abraxane), and the aromatase inhibitor Arimidex. Each of these two drugs were tested on a single cell line, and we create LINCS features for each tumor by combining that tumor’s continuous log-fold differential expression with the expression change induced by that drug in the appropriate cell line. We compute two features for each (tumor,drug) pair:

- Correlation between that tumor’s differential expression and the cell line differential expression induced by administering that drug.
- Dot product between that tumor’s differential expression and the cell line differential expression induced by administering that drug.

While the two features above are conceptually similar, we note that in addition to the direction of agreement, the dot product also represents the *magnitude* of change in expression between a tumor and the cell line in question.

### Clinical feature extraction

We use the following categorical variables from the general TCGA clinical data:

- Tumor pathological stage
- Node pathological stage
- Metastasis pathological stage
- Overall pathological stage
- Histological type
- ICD-10 type
- ICD-O-3 histology
- HER2 immunohistochemistry level result
- Post-surgery margin status

We expand each categorical variable listed above into 0/1 indicator columns for use in classification methods. We additionally extract the estrogen receptor status of each tumor, used for selecting additional patients based on prior clinical knowledge.

### Classification

With the above features, we perform cross-validation experiments to assess our ability to predict response to aromatase inhibitor treatment. We examine all patients who were given any of the aforementioned drugs: 279 patients were given anastrozole, 51 were given exemestane, and 80 were given letrozole. An additional 180 samples were not considered for aromatase inhibitor therapy due to having ER– tumors, which are known not to respond to AI therapy. In this general aromatase inhibitor response prediction task, we assign a patient a “non-response” label if they were removed from any such drug for clinical reasons, or if the patient died during drug treatment. We also include prior clinical knowledge in this “all aromatase inhibitor” analysis; we integrate this prior knowledge by also computing the above features for the 180 patients who were not given an aromatase inhibitor, but who had estrogen receptor negative (ER–) tumors. These tumors are known not to respond to this type of treatment, so we assign these samples “non-response” labels. We use the features discussed above to learn various types of classifiers including logistic regression (with both L1 and L2 regularization), Random Forest and Probabilistic SVMs. For each of these methods and each setting we perform leave-one-out cross-validation.

### Cell line treatment

We performed cell line experiments to compare breast cancer cell line growth with and without the addition of estrogen. We initially grow cell cultures for 5 days, with estrogen present, simulating the initial growth of breast cancer in a patient. We then separately grow cultures with a continued supply of serum estrogen, or with replacement cell medium that lacks estrogen—the environment without estrogen simulates the introduction of an aromatase inhibitor. We measure cell line growth with and without serum estrogen after the initial growth period, and from these cell counts we compute measures of how much each cell line responded to the presence of estrogen. We performed this experiment with 12 replicates of each cell line, 6 with and 6 without estrogen after the initial growth period, and used a mixture of ER+ and ER– cell lines (details in Table B in S1 Text). We computed a growth measure for these cells as

$$\begin{array}{c} \sqrt{\frac{\text{1nM}\;\text{E2}\;\text{GR}-1}{\text{no}\;\text{E2}\;\text{GR}-1}(\text{1nM}\;\text{E2}\;\text{GR}-\text{no}\;\text{E2}\;\text{GR})} \end{array}$$ (6)

with “GR” denoting growth ratio with or without serum E2.

### Cell line experiment

MCF-7, BT474, BT483, CAMA1, Uacc812, ZR75-1 ZR75-30 and T47D breast cancer cell lines were purchased from American Type Culture Collection [ATCC], Manassas, VA, USA. SUM44PE was purchased from Asterand Bioscience, Detroit, MI, USA, and 600MPE cells were a gift by Dr. Rachel Schiff. For the estrogen removal experiments, the cells were kept for 5 days in IMEM supplemented with 10% charcoal stripped serum (CSS) with 1nM E2, and then plated into 96-well plates with or without 1 nM estradiol. An exception are Sum44PE cells that were kept in IMEM with 2% CSS. After 5 days, cell numbers were measured using Cell-titer Glo (Promega, Madison, WI, USA) according to the manufacturer’s instructions. Luminescence was measured with GloMax^®^multi-Detection System (Promega, Madison, WI, USA), using a VICTOR X4 plate reader (PerkinElmer, Waltham, MA, USA). Bars represent the mean of six biological replicates ± SD. 17*β*-Estradiol (E2) was obtained from Sigma-Aldrich (St. Louis, MO, USA).

### Combining cell line and patient derived classifiers

We separated a random 10% of patients to use for training weights between tumor and cell line classifiers, and used the remaining 90% of patients for cross-validation analysis. In each cross-validation fold, we fit classifiers to the corresponding training set of patients, and then used those classifiers to produce non-response predictions of the 10% of patients initially set aside. We then computed predictions for those 10% of patients using classifiers trained on cell lines, and chose the optimal convex combination of tumor and cell line predictions in the training set, producing final prediction *p* = *γp*_*c*_+(1 − *γ*)*p*_*p*_, with *p*_*c*_ denoting predictions from cell lines and *p*_*p*_ denoting predictions from tumors. The validation set predictions then combines the tumor and cell line predictions via the hyper-parameter *γ* tuned by cross-validation (note that in this way we can use the full set of features for tumors while still using the cell lines in the prediction algorithm).

## Supporting information

S1 Text Supporting information. Descriptive statistics of the UPMC patient cohort, details of comparisons with other methods, and results of additional analyses.
(PDF)

S1 Data Analysis scripts and processed data (network-constructed features) used to produce the results shown in this work. (ZIP)
