# Supplementary material for: Network-guided prediction of aromatase inhibitor response in breast cancer
Source: PLoS Comput Biol. 2019 Feb 11;15(2):e1006730. doi: 10.1371/journal.pcbi.1006730 (PMC6386390; doi:10.1371/journal.pcbi.1006730)
Supplement: S1 Text — Descriptive statistics of the UPMC patient cohort, details of comparisons with other methods, and results of additional analyses. (PDF) [file pcbi.1006730.s001.pdf]

Network-Guided Prediction of Aromatase Inhibitor Response in Breast Cancer

(Supplementary Material)

Matthew Ruffalo[1], Roby Thomas[2], Jian Chen[2], Adrian V. Lee[2], Steffi Oesterreich[2], and Ziv Bar-Joseph[1,3]

[1]*Computational Biology Department, School of Computer Science, Carnegie Mellon University, Pittsburgh, PA, USA*

[2]*Women's Cancer Research Center, Department of Pharmacology and Chemical Biology, UPMC Hillman Cancer Center, Magee Womens Research Institute, Pittsburgh, PA, USA*

[3]*Machine Learning Department, School of Computer Science, Carnegie Mellon University, Pittsburgh, PA, USA*

**Demographics**		
Age	58 ± 21	
Post-menopause	96	(64%)
**Stage**		
Stage 0	1	(0.67%)
Stage I	57	(38%)
Stage IA	13	(8.67%)
Stage II	1	(0.67%)
Stage IIA	50	(33.3%)
Stage IIIA	3	(2%)
Stage IIIB	3	(2%)
Stage IIIC	2	(1.33%)
Stage IV	2	(1.33%)
Unavailable	5	(3.33%)
**Outcome**		
Alive at last followup	96	(64%)
Time to death (months)	65.5 ± 39.75	

Table A. UPMC patient characteristics. Aggregate measures are shown as median ± inter-quartile range (IQR).

Cell Line Name	ER Status	PR Status
600MPE	+	−
BT474	+	+
BT483	+	+
CAMA1	+	−
MCF7	+	+
MDAMB175VII	+	−
MDAMB361	+	−
MDAMB415	+	−
SUM44	+	−
T47D	+	+
UACC812	+	−
ZR751	+	−
ZR7530	+	−

Table B. Hormone receptor characteristics for cell lines used in this work. ER denotes estrogen receptor status; PR denotes progesterone receptor status.

Data

UPMC Patients

Table A shows descriptive statistics for the UPMC patient cohort.

Cell Lines

Table B shows information for the cell lines studied in this work.

Methods

Comparison with other methods

Reijm *et al.*

Reijm *et al.* [1] describe an eight-gene classifier for prediction of aromatase inhibitor response, and present t-statistic values for association of these genes with patient response. Though there are conceptual differences between t-statistic values and logistic regression coefficients, we nonetheless can use these t-values to produce continuous predictions of patient response with log-fold gene expression data.

We extract a subset of this gene expression data E with rows as patients and columns as these eight genes, and collect the t-statistic values into a vector τ with matching ordering of genes. Note that Reijm *et al.* denote higher association with

response as higher t-statistic values, whereas we predict *non*-response – we therefore use the additive inverse of their values in the vector τ. We then compute the prediction score p_i for patient i as:

$$p_i = 1/\exp\left(-E_i.\tau\right) \tag{1}$$

Hofree *et al.*, network-based stratification of tumor mutations

We apply Hofree *et al.*'s network-based stratification of tumor mutations [2] to this mutation data, with parameter k chosen to produce two unordered clusters of patients. We evaluate both permutations of these two clusters, and use the numeric cluster assignments $c \in \{1, 2\}$ from the optimal clustering as patient predictions. We see that the optimal permutation of clusters is not particularly informative for either prediction of response to all aromatase inhibitors or anastrozole specifically (ROC AUC 0.5316 and 0.5630, respectively).

Leiserson *et al.*, WExT mutational exclusivity

We also use Leiserson *et al.*'s WExT method [3] to identify sets of significantly mutually exclusive mutations across the entire patient cohort. Genes common to the same pathway often show mutual exclusivity in somatic mutations [4], and identification of such gene sets shows promise in *de novo* pathway identification [5]. We therefore treat gene sets identified by WExT with significant mutational exclusivity ($P < 0.002$ as reported by the tool) as potential pathways. For each of the 18 such gene sets, we compute a Boolean vector for our patient cohort, with 1 denoting that the patient has a somatic mutation in that gene set, and 0 otherwise. This produces a binary gene set membership matrix G with rows as patients and 18 columns, corresponding to these exclusive gene sets. We compute the row-wise sum of this matrix, assigning each patient a score $\in [0, 18]$ equal to the number of putative *de novo* pathways that are mutated in that patient. We hypothesize that a higher mutational load will correspond with drug non-response, and indeed this "WExT Mutation Set Count" score is reasonably informative in prediction of non-response to anastrozole (ROC AUC 0.6212), though is less informative in the "all aromatase inhibitor" prediction task (ROC AUC 0.5509).

Wang *et al.*, similarity network fusion

We apply Wang *et al.*'s similarity network fusion [6] method to our data, using a concatenation of the binary somatic mutation matrix M and the log-fold expression matrix E. We use parameters suggested in the SNF paper and package documentation: number of neighbors $K = 20$, affinityMatrix hyperparameter $\alpha = 0.5$, SNF iterations $T = 20$. As in SNF example code, we perform spectral clustering on the SNF results to separate the samples into two groups. As in our usage of Hofree *et al.*'s NBS method, we use the group assignments as class labels, and select the optimal permutation of these class labels as response predictions for samples.

Results

Predicting aromatase inhibitor response for all TCGA patients

Figure A shows univariate feature ROC AUC for aromatase inhibitor non-response, using raw feature values for computation of this AUC measure. Figures B, C, and D show genes' contributions to PCA component features for those scoring highly in the random forest feature importance measure described in the main text.

Performance improvement from feature construction and UPMC clinical data

Figures E and F show the performance of non-response prediction cross-validation with the "naïve" feature set described in the main text, for all aromatase inhibitors and anastrozole, respectively. Figures I, J, and K show cross-validation performance with only UPMC patients, for all aromatase inhibitors with constructed features (I), all aromatase inhibitors with "naiïve" features (J), and anastrozole with "naïve" features (K). Figures G and H likewise show the results of cross-validation using the "nai|ïve" feature set, transformed via PCA, using 8, 32, 128, and 512 PCA components as features.

Cell Line Experiments

Figure L shows our predictions of cell line non-response to serum estrogen, using classifiers trained on patient response to aromatase inhibitors. Figure M shows correlation between these predictions and the cell line growth measure defined in the main text.

Survival by High-Scoring Genes

Figure N shows Kaplan-Meier plots of patient survival, with patients individually stratified by three genes of interest: Na shows *TP53*, Nb shows *CDH1*, and Nc shows *CCND1*. Figure O likewise shows joint stratification of patients by mutation status in *TP53*, *CDH1*, and *CCND1*. Log-rank P-values for each stratification of patients are shown in the bottom-right of each plot.

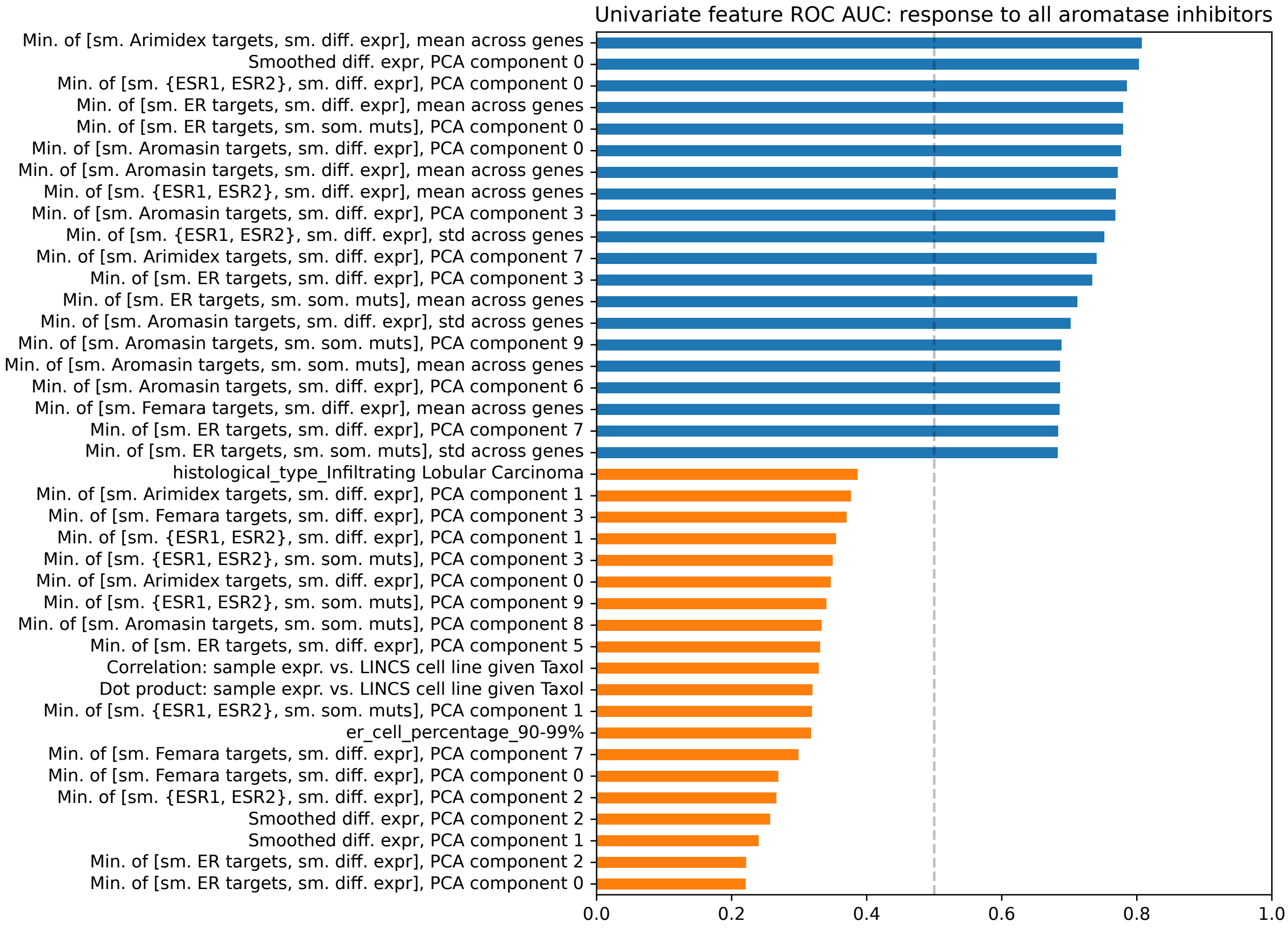

Figure A. Univariate feature ROC AUC for aromatase inhibitor non-response, showing omic features, indicator columns for categorical clinical features, and PCA-decomposed features. Features prefixed with "Min." denote elementwise minimum of pairs of matrices, *e.g.* smoothed drug targets of Arimidex and smoothed binary differential expression as shown in the first feature listed. These (sample, gene) matrices are collapsed across genes in various ways to produce feature values for samples: mean or standard deviation across all genes, or through PCA decomposition. Categorical clinical features are represented with one-hot encoding, and are shown as "feature name_column name", *e.g.* "er_cell_percentage_90-99%". The dashed line shows ROC AUC 0.5.

Combining Cell Line and Patient Derived Classifiers

Figure P shows the results of combining cell line and patient-derived classifiers; Pa shows cross-validation results from the combined classifier, and Pb shows the selection of the tuning parameter γ, which selects the contribution of cell line classifiers.

Comparison with other methods

Figure Q shows a consolidated ROC plot for non-response to all aromatase inhibitors, including additional variants of our analysis that are not shown in Figure 5. Figure R shows a similar ROC plot specifically for non-response to anastrozole, including only samples which were administered that drug.

References

1. Reijm EA, Sieuwerts AM, Smid M, Bolt-de Vries J, Mostert B, Onstenk W, et al. An 8-gene mRNA expression profile in circulating tumor cells predicts response to aromatase inhibitors in metastatic breast cancer patients. BMC cancer. 2016;16(1):123.

2. Hofree M, Shen JP, Carter H, Gross A, Ideker T. Network-based stratification of tumor mutations. Nature Methods. 2013;10(11):1108–1115. doi:10.1038/nmeth.2651.

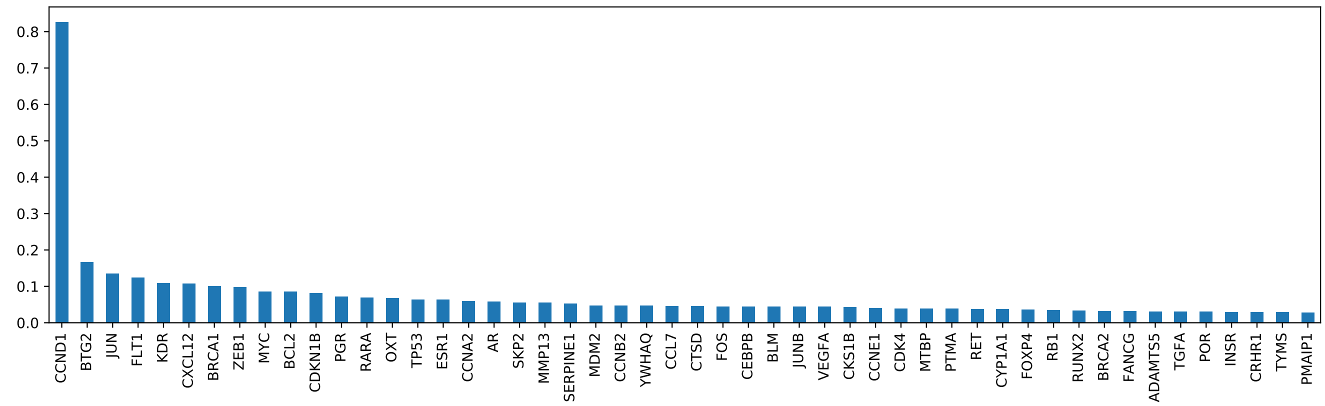

Figure B. Gene scores for PCA component 2 of estrogen receptor target/smoothed differential expression minimum matrices. Genes are sorted by absolute value of PCA component score.

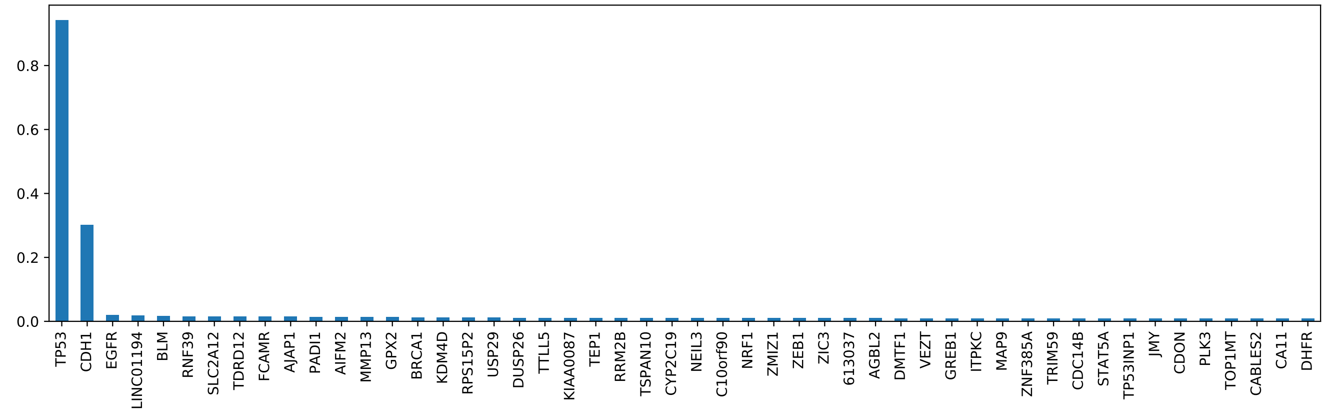

Figure C. Gene scores for PCA component 0 of estrogen receptor target/smoothed mutation minimum matrices. Genes are sorted by absolute value of PCA component score.

3. Leiserson MD, Reyna MA, Raphael BJ. A weighted exact test for mutually exclusive mutations in cancer. Bioinformatics. 2016;32(17):i736–i745.

4. Szczurek E, Beerenwinkel N. Modeling mutual exclusivity of cancer mutations. PLoS Comput Biol. 2014;10(3):e1003503.

5. Ciriello G, Cerami E, Sander C, Schultz N. Mutual exclusivity analysis identifies oncogenic network modules. Genome research. 2012;22(2):398–406.

6. Wang B, Mezlini AM, Demir F, Fiume M, Tu Z, Brudno M, et al. Similarity network fusion for aggregating data types on a genomic scale. Nature methods. 2014;11(3):333.

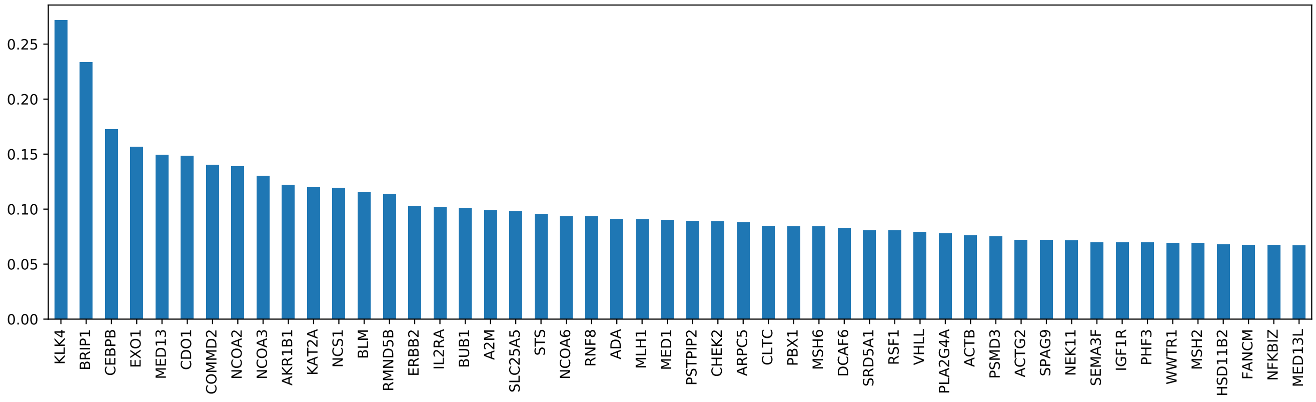

Figure D. Gene scores for PCA component 4 of aromasin target/smoothed differential expression minimum matrices. Genes are sorted by absolute value of PCA component score.

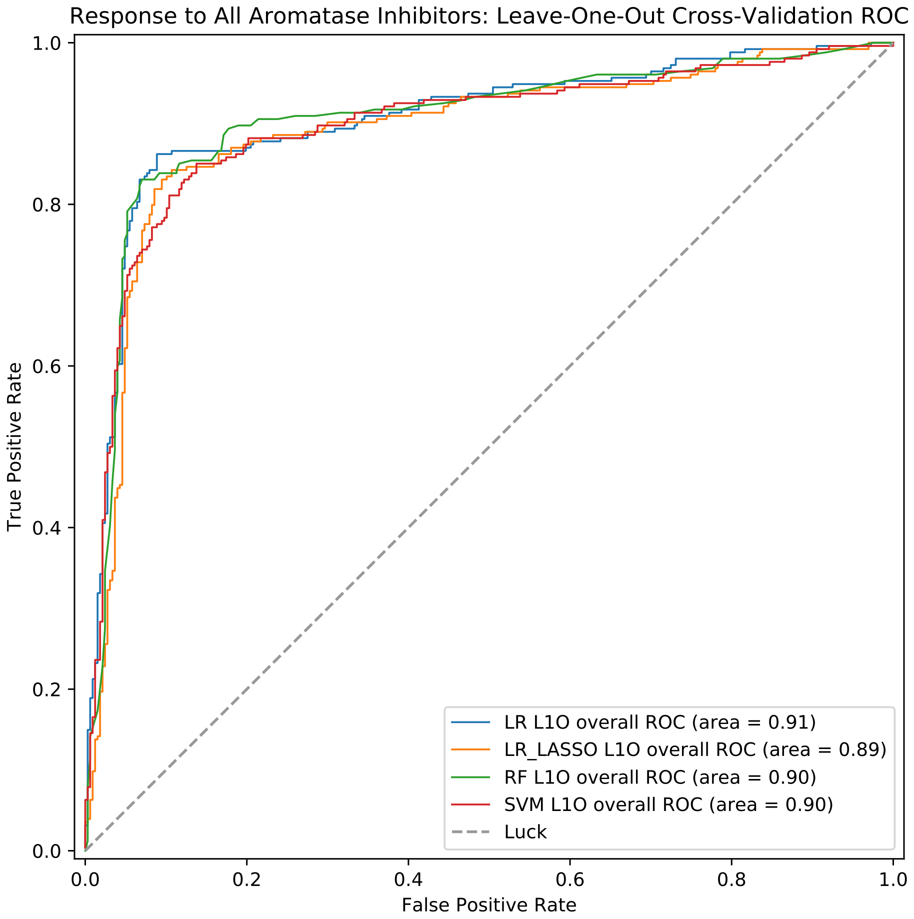

Figure E. Leave-one-out cross-validation prediction results for aromatase inhibitor non-response, using the “naïve” binary feature set.

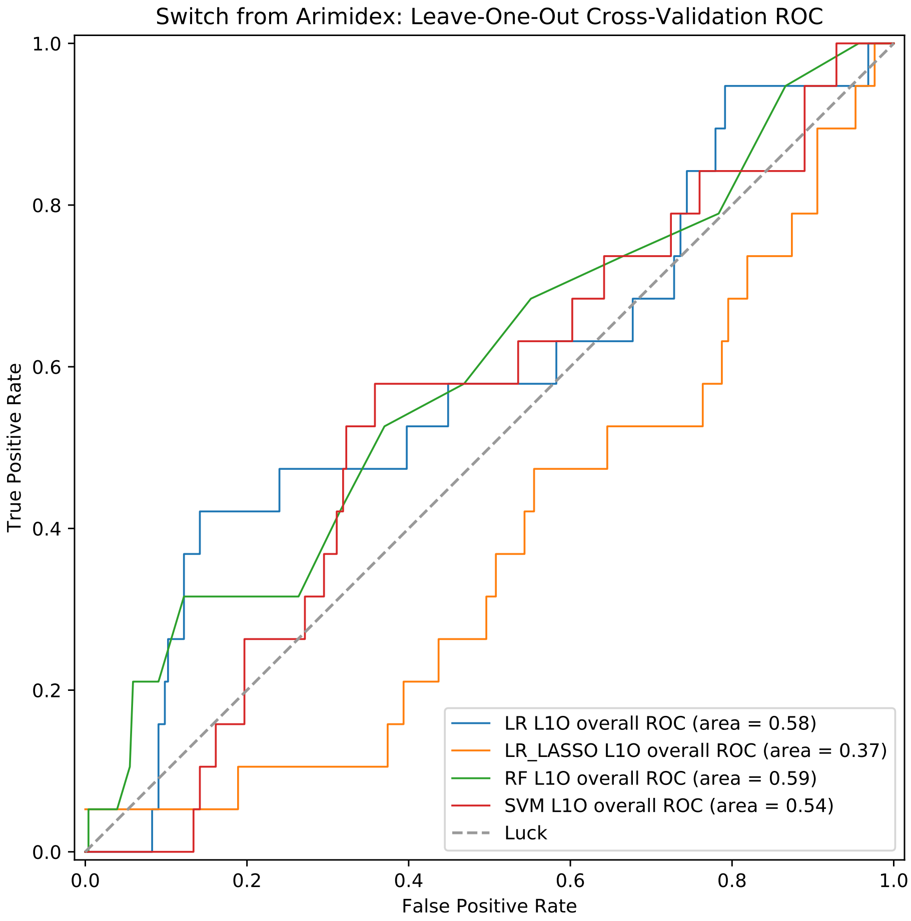

Figure F. Leave-one-out cross-validation prediction results for non-response to anastrozole, using the “naïve” binary feature set.

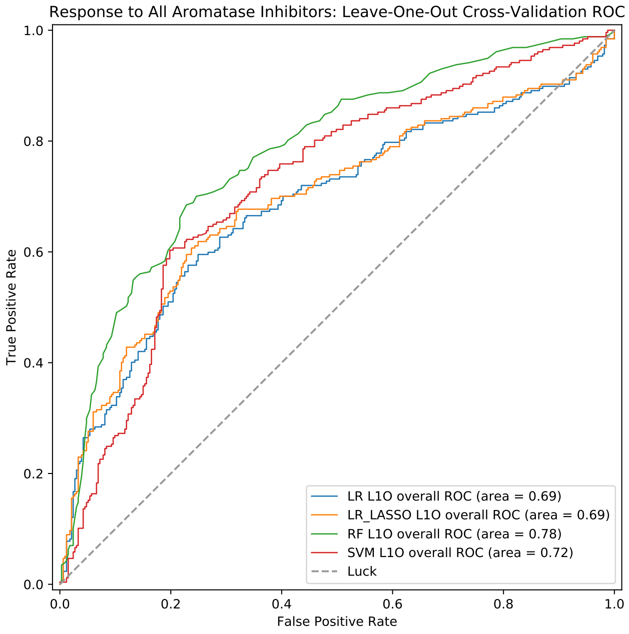

(a) 8 PCA components.

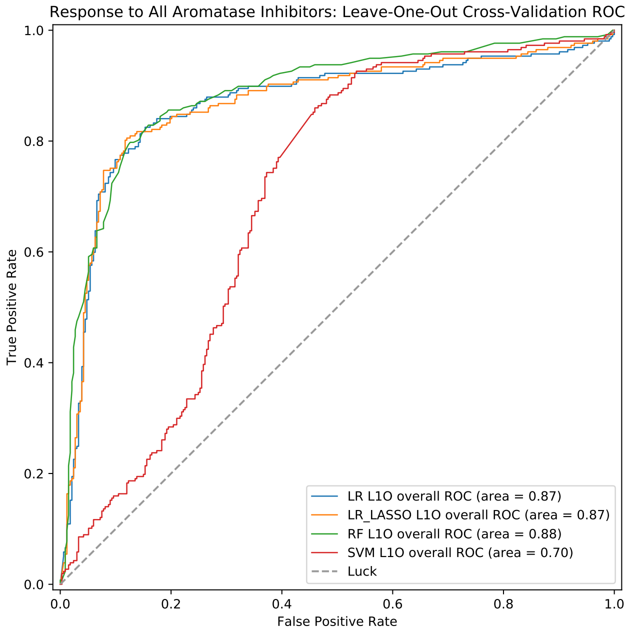

(b) 32 PCA components.

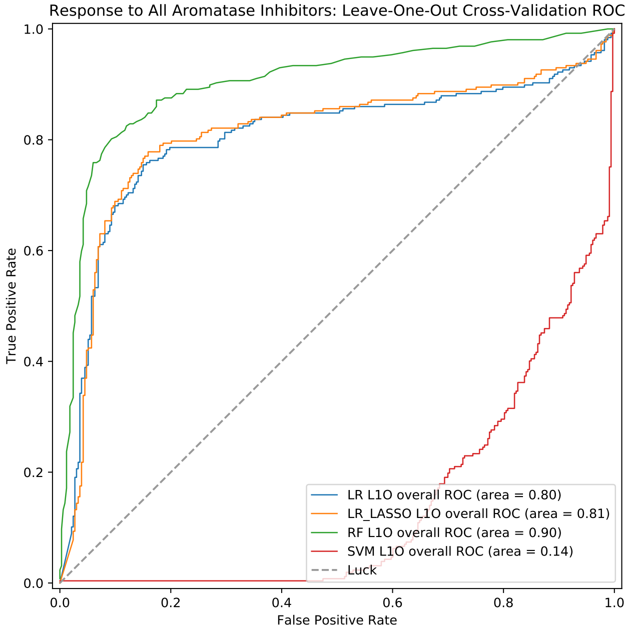

(c) 128 PCA components.

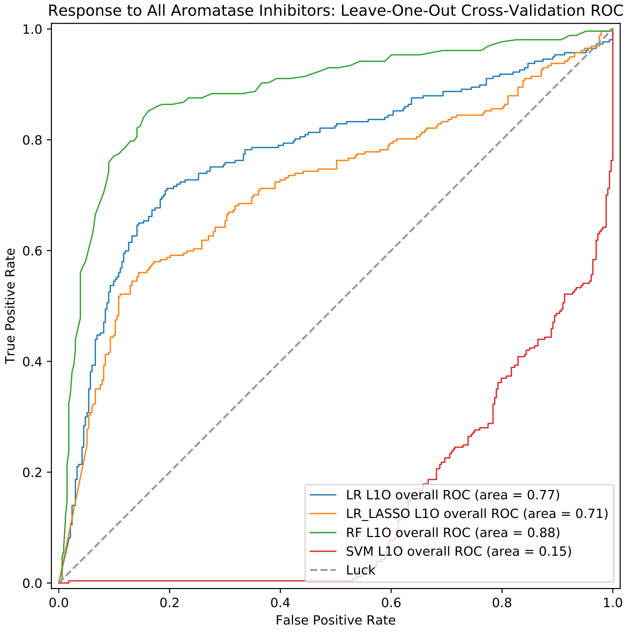

(d) 512 PCA components.

Figure G. Leave-one-out cross-validation prediction results for aromatase inhibitor non-response, using PCA transformations of the “naïve” binary feature set.

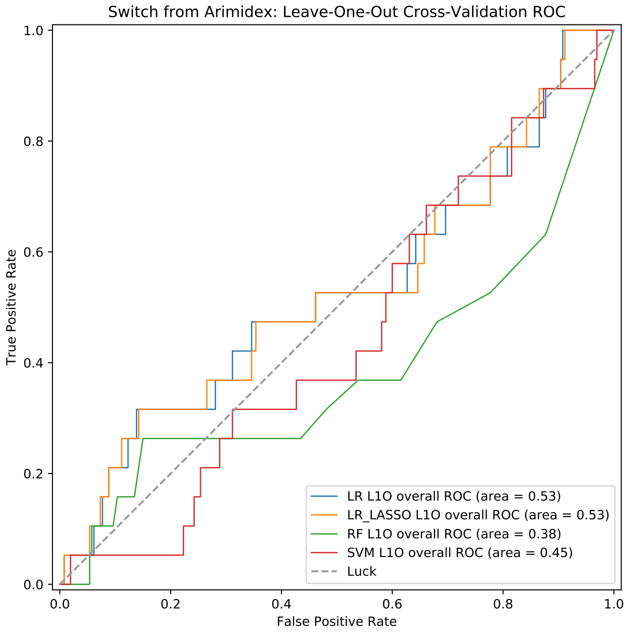

(a) 8 PCA components.

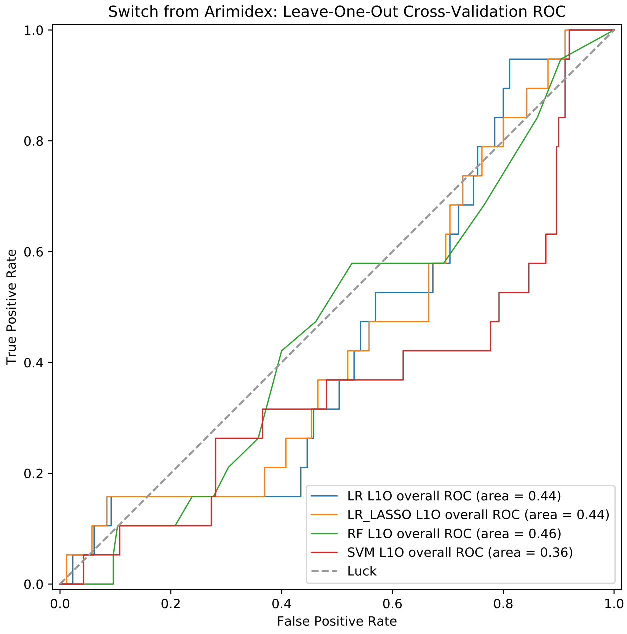

(b) 32 PCA components.

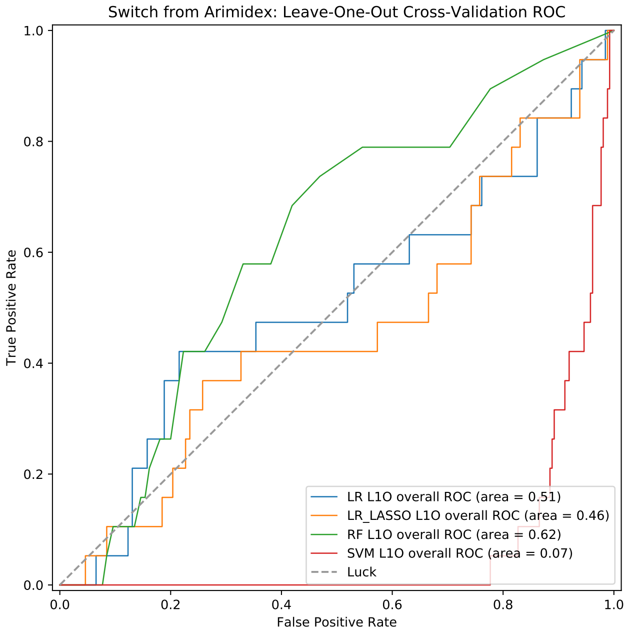

(c) 128 PCA components.

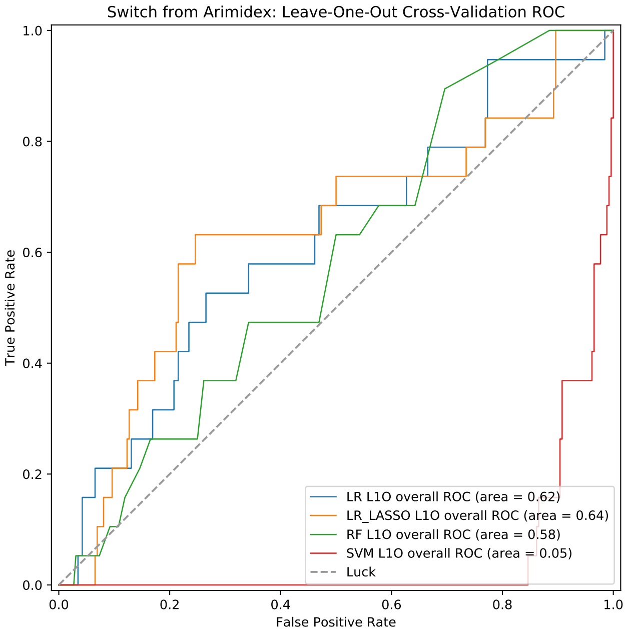

(d) 512 PCA components.

Figure H. Leave-one-out cross-validation prediction results for non-response to anastrozole, using PCA transformations of the “naïve” binary feature set.

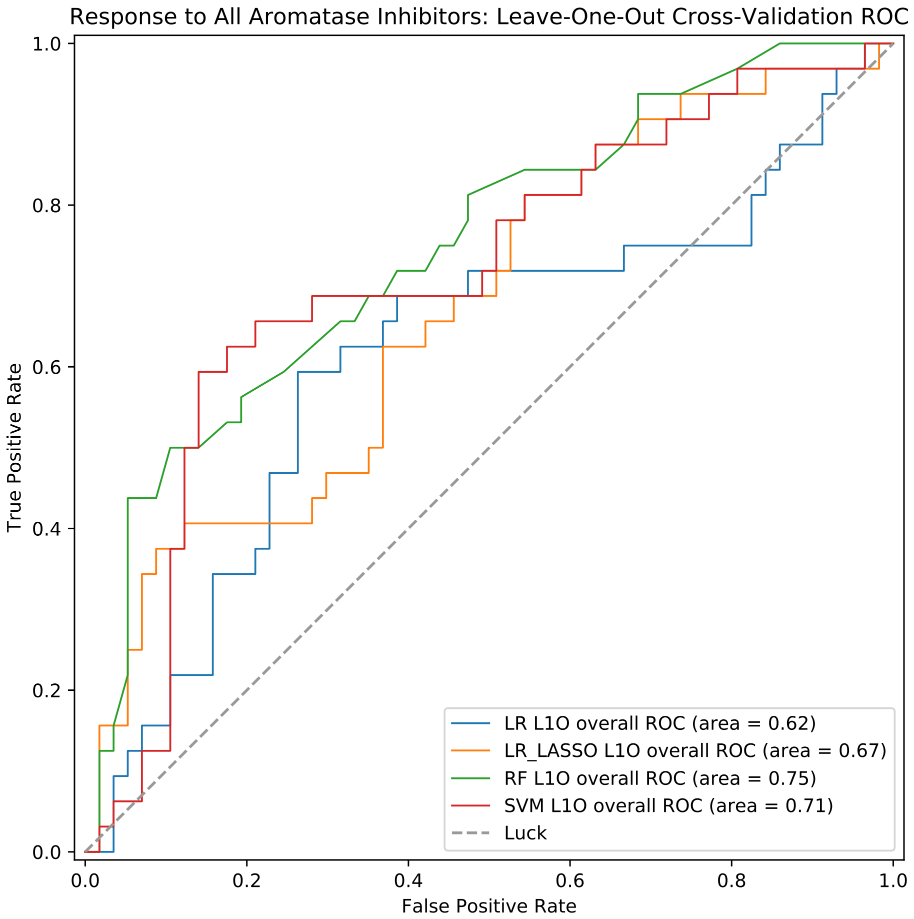

Figure I. Cross-validation prediction results for aromatase inhibitor non-response, restricted to UPMC patients.

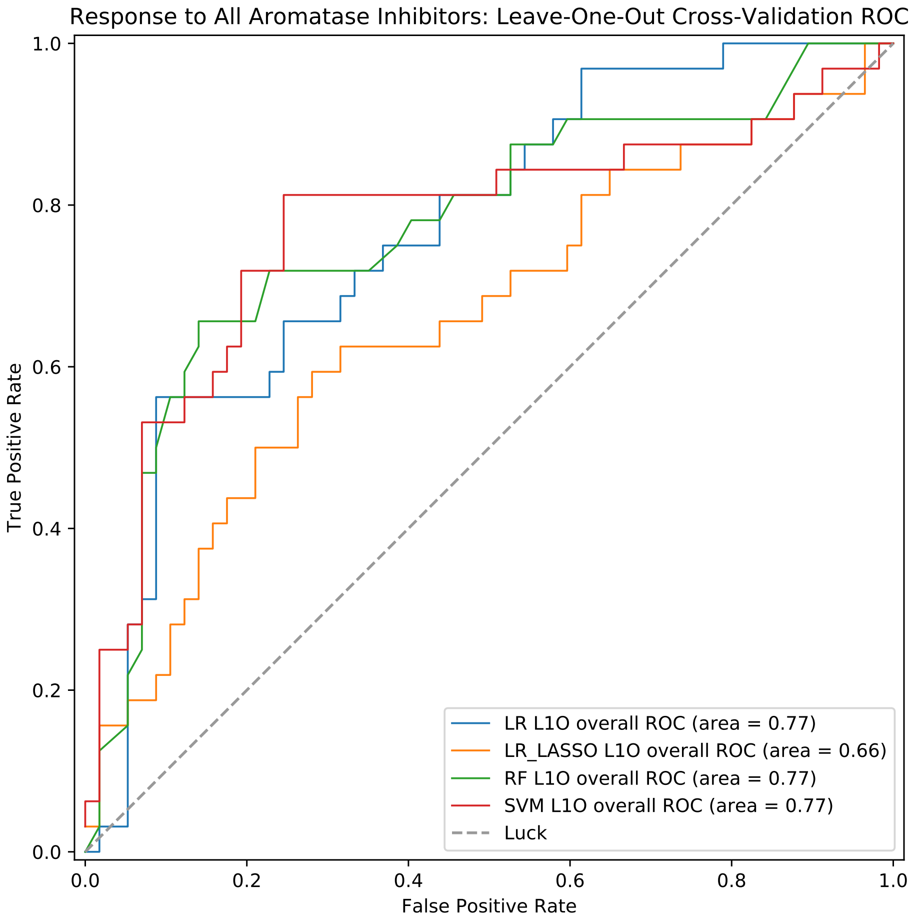

Figure J. Cross-validation prediction results for aromatase inhibitor non-response, restricted to UPMC patients, using the "naïve" binary feature set.

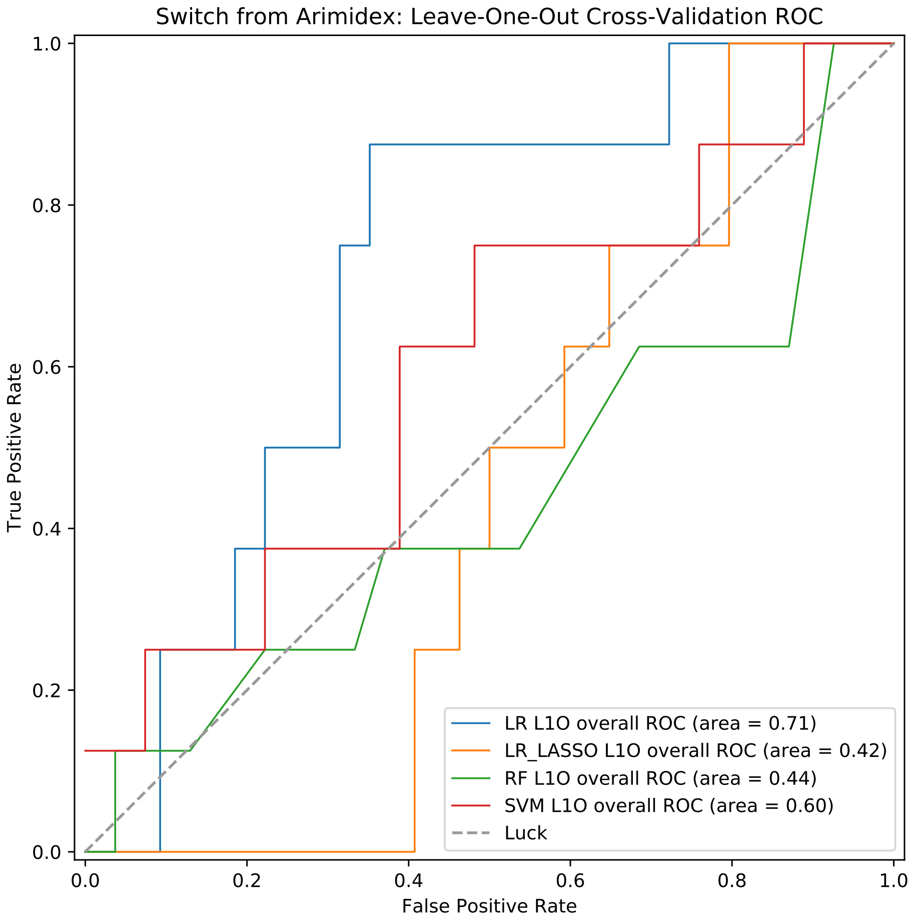

Figure K. Cross-validation prediction results for non-response to anastrozole, using random forests, restricted to UPMC patients, and with the "naïve" binary feature set.

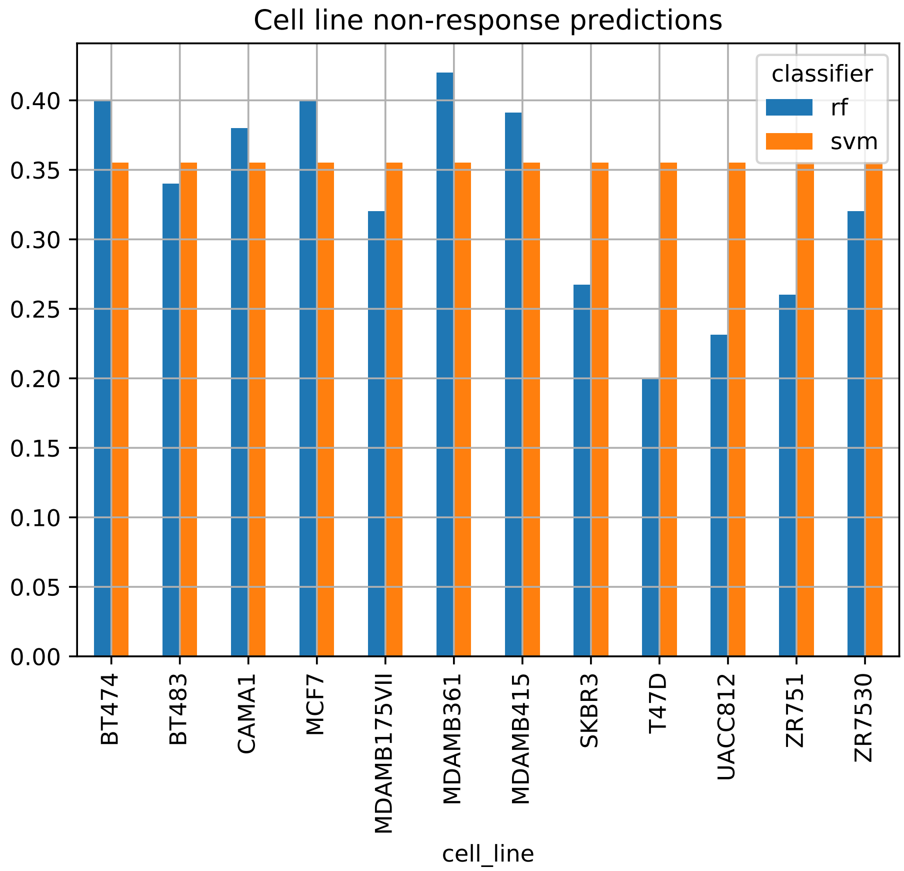

Figure L. Predictions of cell line non-response to serum estrogen, using classifiers trained on patient response to aromatase inhibitors.

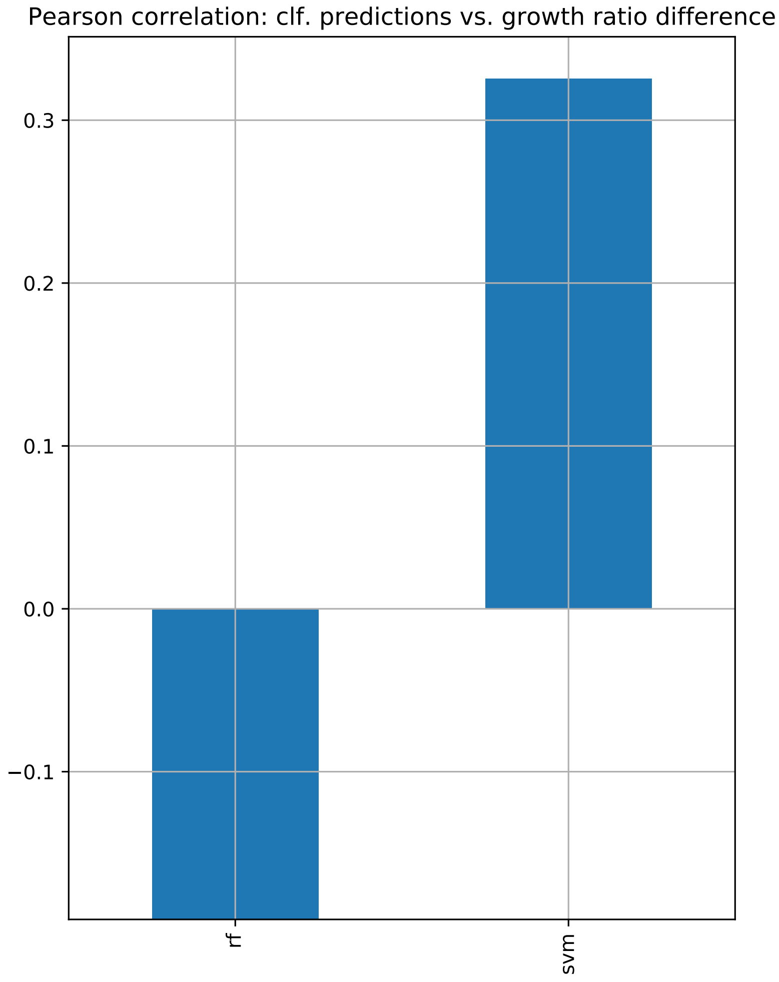

Figure M. Correlation between cell line non-response predictions and reciprocal of the growth measure.

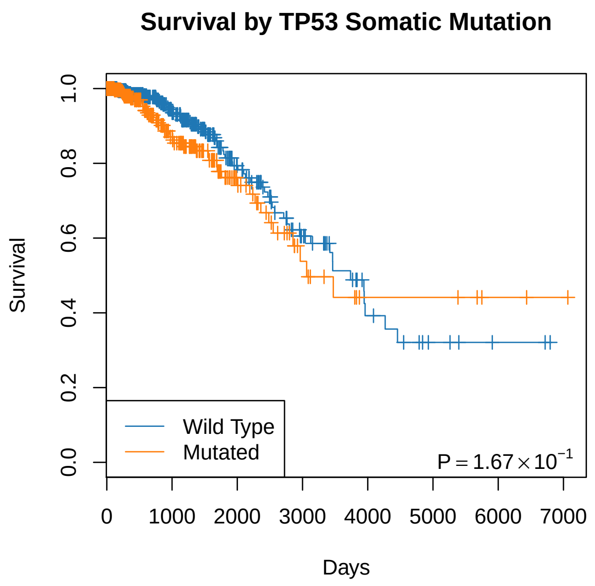

(a) Kaplan-Meier curves of patient survival, stratified by *TP53* mutation status.

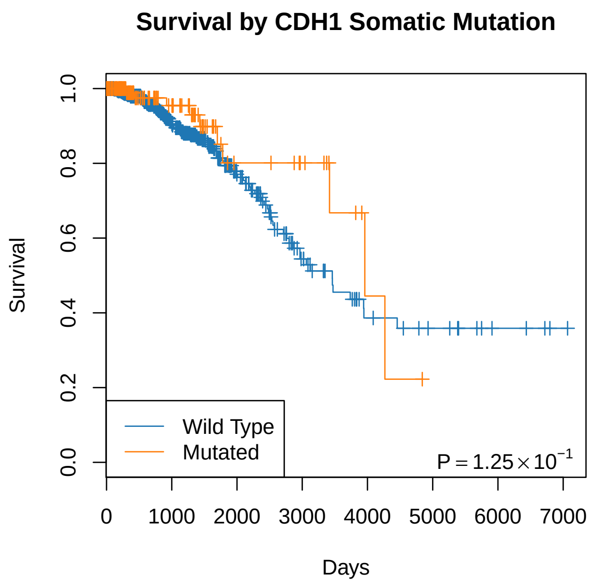

(b) Kaplan-Meier curves of patient survival, stratified by *CDH1* mutation status.

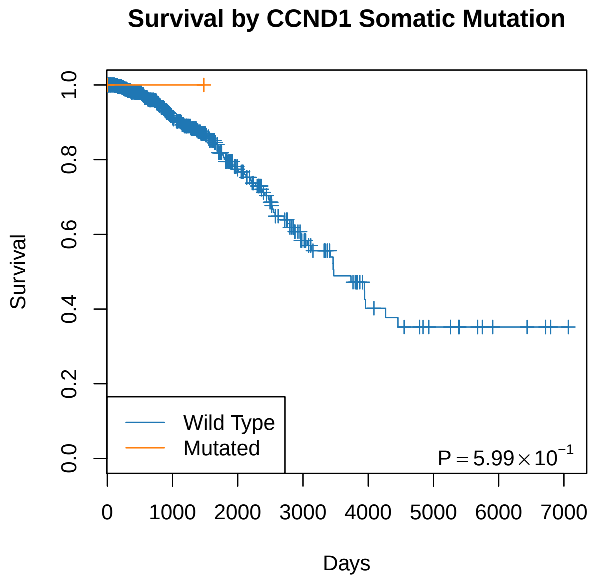

(c) Kaplan-Meier curves of patient survival, stratified by *CDND1* mutation status.

Figure N. Kaplan-Meier curves of patient survival, individually stratified by mutation status in genes identified through high contribution to important PCA features. The log-rank P-value for each stratification of patients is shown in the bottom-right of each plot.

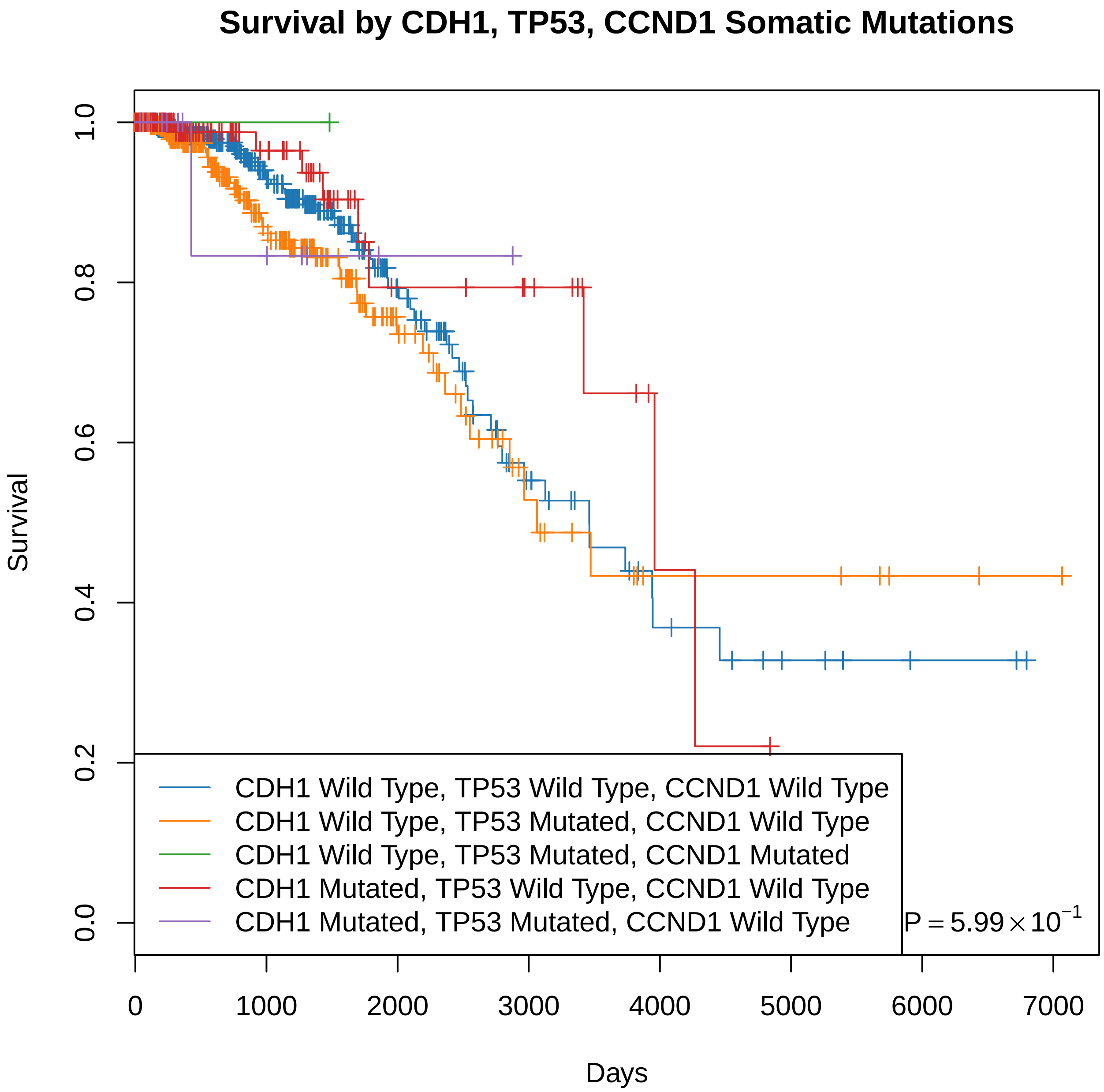

Figure O. Kaplan-Meier curves of patient survival, jointly stratified by mutation status in genes identified through high contribution to important PCA features. The log-rank P-value for this joint stratification of patients is shown in the bottom-right of the plot.

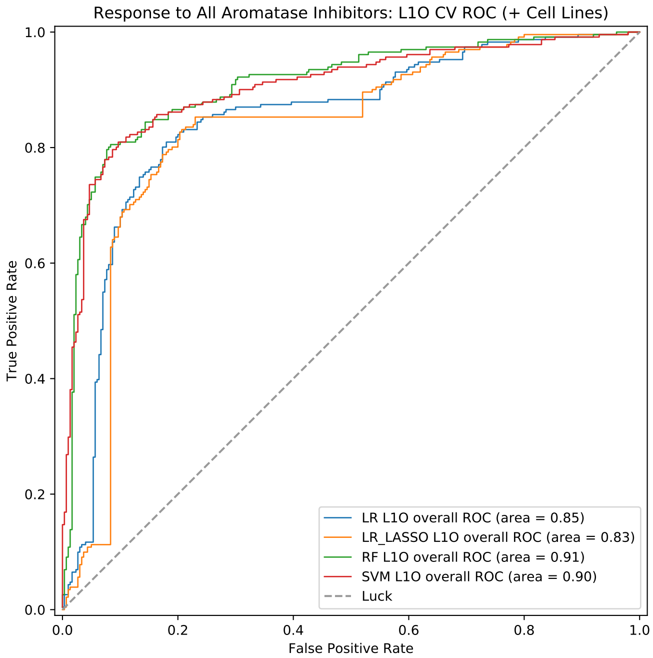

(a) Cross-validation results from the dual model fit to cell lines and patients, with selection of the tuning parameter γ performed on training set samples.

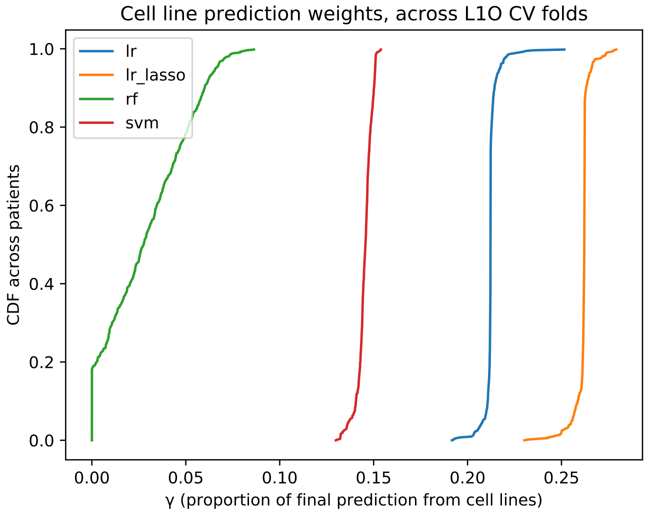

(b) Parameters of the dual models fit to cell lines and patients across leave-one-out cross-validation folds, showing cumulative distribution curves for optimal values of the tuning parameter γ, which selects the convex combination of cell line and patient predictions in the training set.

Figure P. Results for combining cell line and patient-trained classifiers.

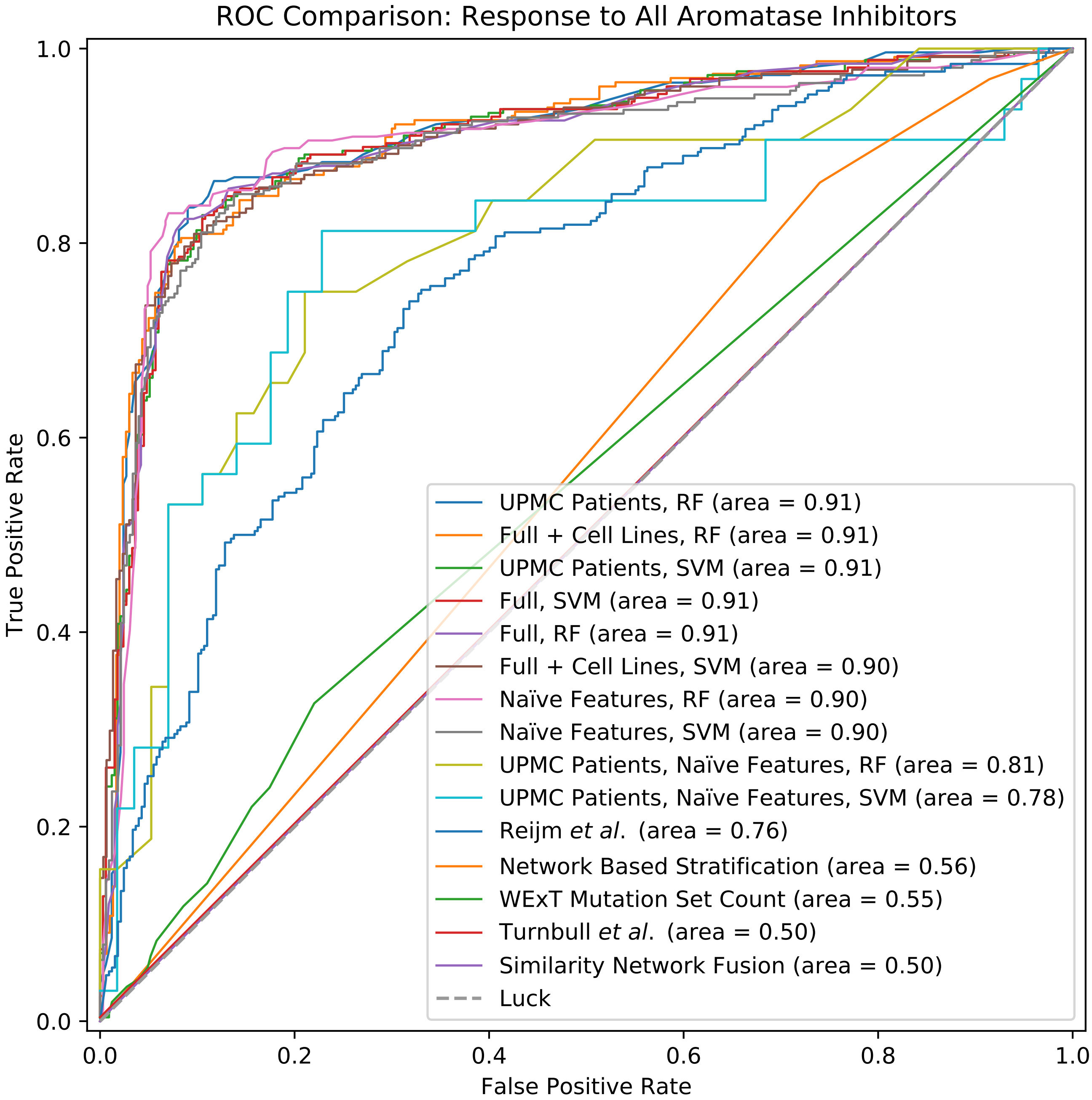

Figure Q. Performance comparison between all prediction strategies, for prediction of non-response to all aromatase inhibitors.

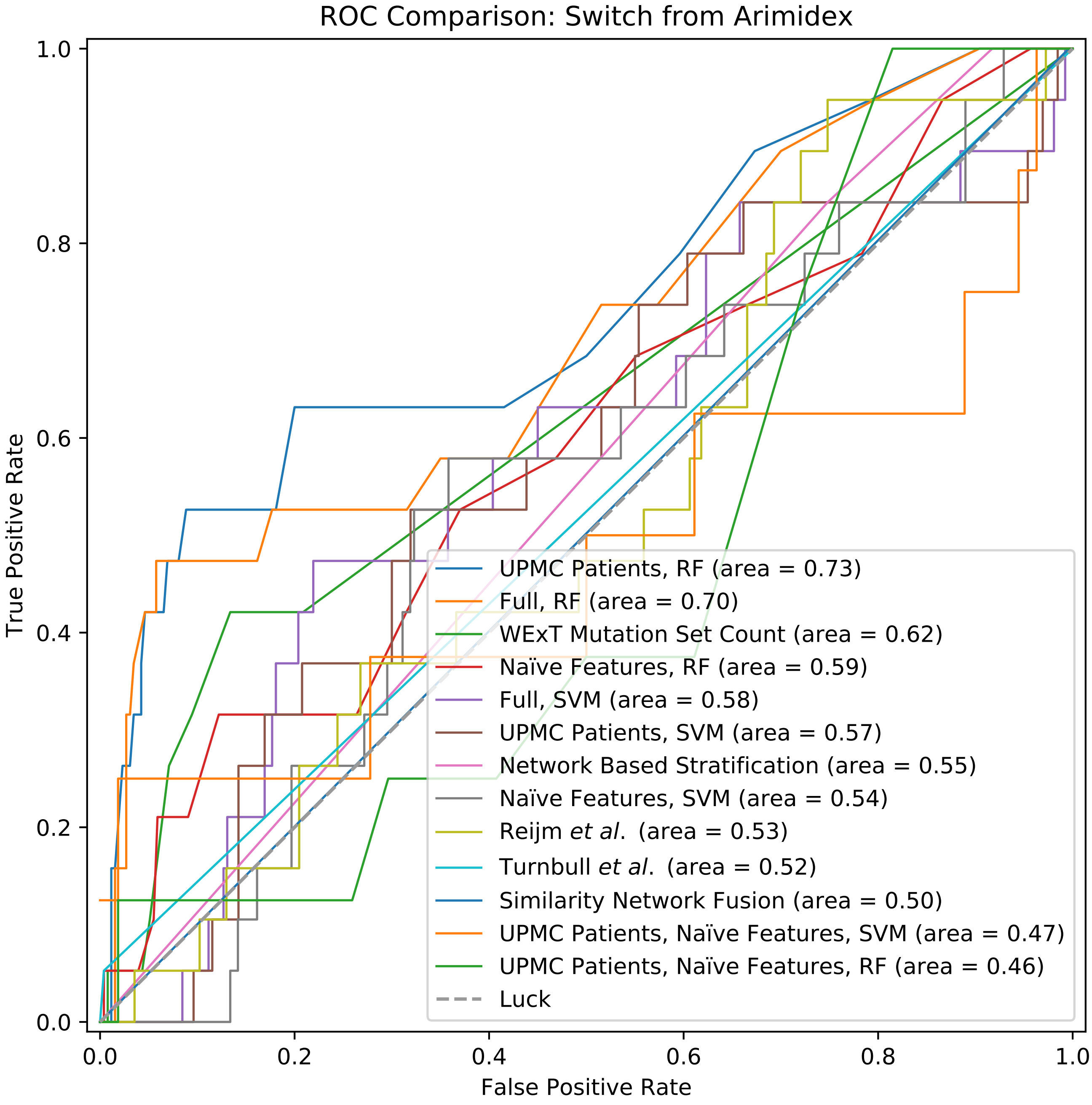

Figure R. Performance comparison between all prediction strategies, for prediction of non-response to anastrozole.
